# Immune–metabolic convergence in skeletal remodeling disorders: shared osteoimmune, senescent and lipid mechanisms across aging-related and immune-mediated bone disease

**DOI:** 10.3389/fimmu.2026.1949165

**Published:** 2026-09-25

**Authors:** Ting-ting Guo, Hong-Xin Zheng, Qing-dan Jiang

**Affiliations:** Liaoning University of Traditional Chinese Medicine, Shenyang, China

**Keywords:** ferroptosis, immunosenescence, inflammaging, lipid metabolism, osteoarthritis, osteoimmunology, osteoporosis, RANKL

## Abstract

Bone and joint disorders of the aging musculoskeletal system are conventionally treated as separate clinical entities. This disease-centered view aids diagnosis but obscures the immune, inflammatory, senescent and metabolic programs they share. This Review compares five of them, grouped by etiology rather than by age: two aging-driven remodeling disorders, osteoporosis (OP) and osteoarthritis (OA); two immune-mediated arthritides in which aging modifies rather than causes the skeletal outcome, rheumatoid arthritis (RA) and psoriatic arthritis (PsA); and one focal, largely monogenic disorder included as a boundary case, Paget’s disease of bone (PDB). RA and PsA are not treated as age-related diseases; they are included because they show most directly that immune activation alone can redirect skeletal remodeling. The review is narrative and mechanism-oriented, based on a structured, auditable search (Section 2); we compare evidence across diseases rather than claim exhaustive synthesis. Skeletal aging, immunosenescence, inflammaging and lipid dysregulation converge on five nodes: the receptor activator of nuclear factor-κB ligand (RANKL)–osteoprotegerin (OPG) axis, the interleukin-17/tumor necrosis factor-α/interleukin-6 network, senescence-associated secretory phenotype (SASP) amplification, phospholipid peroxidation and ferroptosis, and Wnt–sclerostin–Dickkopf-1 (DKK-1) balance. Across diseases these nodes generate different skeletal outputs: systemic bone loss in OP; cartilage degeneration with subchondral remodeling in OA; erosion with impaired repair in RA; erosion combined with ectopic bone formation in PsA; and focal high-turnover remodeling in PDB. Disease identity, we argue, is set by the sign and relative weighting of node activity rather than by which nodes are engaged; the opposing direction of DKK-1 regulation in RA and PsA is the clearest example. Ferroptotic injury is resolved by cell type rather than treated as uniformly harmful, and PDB is retained as a comparator showing that shared-node activation is permissive rather than deterministic. Candidate biomarkers and pathway-directed therapies are summarized with their translational gaps. Every disease × mechanism combination is graded on a four-level evidence scheme (E1, established human evidence, to E4, indirect or *in vitro* only) defined in Section 2. A cross-disease, mechanism-informed approach may improve risk stratification, identify treatable endotypes, and guide future trials in immune-metabolic skeletal disease.

## 1 Introduction

Population aging has made degenerative and metabolic bone diseases a major public-health issue. Osteoarthritis (OA) and osteoporosis (OP) are the two most prevalent aging-driven skeletal disorders; rheumatoid arthritis (RA) and psoriatic arthritis (PsA) add an immune-mediated route to the same skeletal endpoints, and Paget’s disease of bone (PDB) provides a focal, largely genetic comparator. Selection criteria for these five disorders are stated in Section 1.1. The Global Burden of Disease 2021 study estimated that around 595 million people worldwide were living with OA, and projected continued growth in case numbers through 2050 (1). A systematic review and meta-analysis based on the WHO diagnostic criteria reported a global OP prevalence of approximately 18.3% in adults aged 50 years and over, with women affected substantially more often than men (2). PDB has been declining in many regions but remains relatively common in older adults of European descent, with reported prevalence ranging from 1.5% to 8.3% (3). RA and PsA are not defined by chronological age, but their skeletal burden is expressed largely in middle-aged and older adults, in whom reduced repair capacity and coexisting age-related bone loss modify the structural consequences of the same inflammatory drivers (4, 5). These conditions overlap mechanistically; chronic low-grade inflammation and disrupted bone remodeling are recurring features across all five diseases (6).

OA has traditionally been viewed as a wear-and-tear joint disease, OP has been attributed mainly to estrogen deficiency and disturbed calcium-phosphate homeostasis, and PDB has been described as a focal disorder of bone remodeling driven by abnormal osteoclast activity, while RA and PsA have been framed as autoimmune or immune-mediated joint diseases in which skeletal damage is a downstream consequence of synovial and entheseal inflammation. Accumulating evidence suggests these frameworks do not fully capture the clinical heterogeneity and progression of these diseases (7). A recent review in *Nature Reviews Rheumatology* discussed how obesity contributes to OA pain through mechanisms beyond joint loading, including adipose-tissue dysfunction, systemic inflammation and metabolic syndrome (8). In PDB, *SQSTM1* mutations are the main known genetic risk factor, occurring in around 30% of familial cases and a subset of sporadic cases; however, incomplete penetrance and the focal distribution of lesions imply that environmental and microenvironmental factors are likely required for clinical expression (9). The evidence taken together makes it difficult to view these conditions as the product of a single cell type or a single pathway acting in isolation.

Osteoimmunology has developed rapidly as an interdisciplinary field over the past decade. Osteoclasts and immune cells (T cells, B cells and myeloid cells) share the receptor activator of nuclear factor-κB ligand (RANKL)/RANK/osteoprotegerin (OPG) signaling axis, several tumor necrosis factor (TNF)-family ligands and many cytokine receptors, forming a bidirectional regulatory network in the bone marrow microenvironment (10). In aging rat and mouse models, senescent macrophages and neutrophils accumulating in the bone marrow secrete grancalcin (GCA), which suppresses osteogenic differentiation of bone marrow mesenchymal stromal cells while promoting adipogenic differentiation and contributes to skeletal aging (11). In OA, a shift of synovial macrophages towards an M1-skewed phenotype has been linked to synovitis, cartilage degradation and abnormal subchondral bone remodeling; the M1/M2 ratio has been proposed as a potential therapeutic target, although translation is constrained by difficulties in subtype-specific targeting (12). The gut–bone axis is another area with growing support, suggesting that the gut microbiota may indirectly modulate bone metabolism through short-chain fatty acids and immune regulation, although causality in humans is still being established (13). The main skeletal–immune regulatory modules are summarized in **Figure 1**.

**Figure 1:**
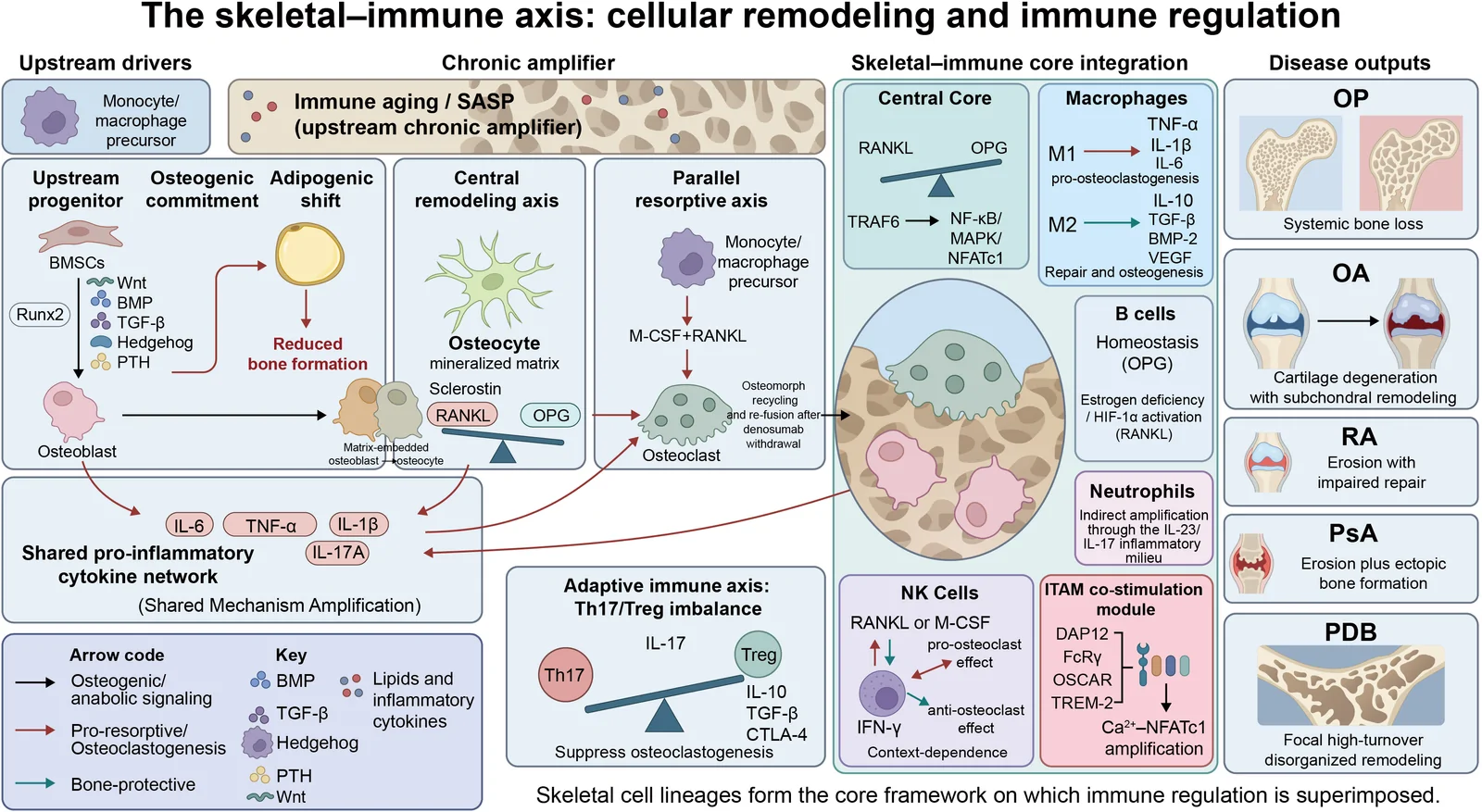
The skeletal–immune axis: cellular remodeling and immune regulation. The schematic summarizes how osteogenic commitment, osteocyte-derived remodeling cues, monocyte/macrophage precursors, adaptive immune imbalance, and innate immune modules interact within the aging bone microenvironment. Immune aging and SASP act as chronic amplifiers, shifting BMSC fate toward adipogenesis, increasing pro-inflammatory cytokine signaling, enhancing osteoclastogenesis, and reducing bone formation. The central RANKL/OPG axis integrates signals from osteocytes, osteoblast-lineage cells, macrophages, B cells, neutrophils, NK cells, and ITAM-associated co-stimulatory pathways, producing disease-specific skeletal phenotypes. BMP, bone morphogenetic protein; BMSC, bone marrow stromal/stem cell; CTLA-4, cytotoxic T-lymphocyte-associated protein 4; DAP12, DNAX-activating protein of 12 kDa; FcRγ, Fc receptor common γ chain; HIF-1α, hypoxia-inducible factor-1α; IFN-γ, interferon-γ; IL, interleukin; ITAM, immunoreceptor tyrosine-based activation motif; M-CSF, macrophage colony-stimulating factor; MAPK, mitogen-activated protein kinase; NFATc1, nuclear factor of activated T cells cytoplasmic 1; NF-κB, nuclear factor-κB; NK, natural killer; OA, osteoarthritis; OP, osteoporosis; OPG, osteoprotegerin; OSCAR, osteoclast-associated receptor; PDB, Paget’s disease of bone; PsA, psoriatic arthritis; PTH, parathyroid hormone; RA, rheumatoid arthritis; RANKL, receptor activator of nuclear factor-κB ligand; Runx2, runt-related transcription factor 2; SASP, senescence-associated secretory phenotype; TGF-β, transforming growth factor-β; Th17, T helper 17 cell; TNF-α, tumor necrosis factor-α; Treg, regulatory T cell; TREM-2, triggering receptor expressed on myeloid cells 2.

Building on this evidence, we propose an integrative framework that links aging, low-grade inflammation, immune dysregulation, lipid-metabolic disturbance and abnormal bone metabolism along a single axis (**Figure 2**). The underlying assumption is that the five disorders considered here, despite different clinical presentations and different initiating causes, converge on a shared set of immune-metabolic regulatory nodes, and that they differ in the sign and relative weighting of activity at those nodes rather than in which nodes are engaged (Section 1.2). The “inflammaging” concept proposed by Franceschi and colleagues, which connects age-related sterile low-grade inflammation with metabolic dysregulation, provides a conceptual basis for this assumption (14). In OA, leakage of mitochondrial DNA into the cytosol of chondrocytes activates the stimulator of interferon genes (STING) pathway and engages nuclear factor-κB (NF-κB) signaling, leading to chondrocyte senescence, apoptosis and extracellular matrix degradation; these findings link cellular stress, senescence and immune activation at the molecular level (15). Obesity and adipose-tissue dysfunction further connect metabolic and immune disturbances through pro-inflammatory adipokines and reprogrammed immune cells, with downstream effects on the bone and joint microenvironment (8). On the translational side, a phase 2 randomized controlled trial of intermittent senolytic therapy (dasatinib plus quercetin, D+Q) in postmenopausal women did not meet its primary bone-resorption endpoint, although a subgroup with a high senescent-cell burden did respond; to avoid repetition the trial is described once, in Section 4.4, and is not restated here (16). The clinical utility of this framework remains to be tested in further prospective studies, particularly in biomarker discovery and the identification of shared cross-disease therapeutic targets.

**Figure 2:**
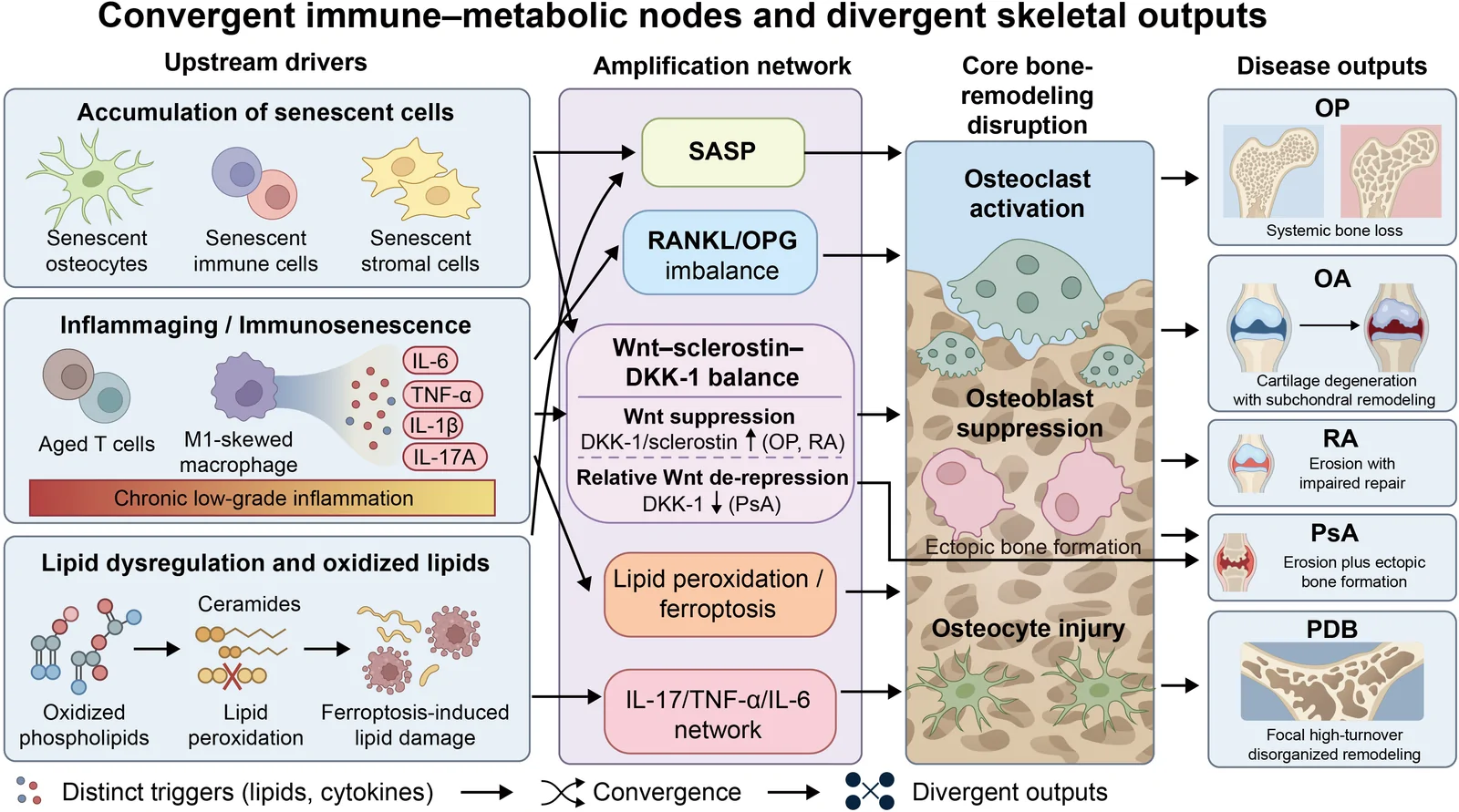
Convergent immune–metabolic nodes and divergent skeletal outputs. Accumulation of senescent cells, inflammaging and immunosenescence, and lipid dysregulation with oxidized phospholipid generation act as upstream drivers that converge on a shared amplification network: senescence-associated secretory phenotype (SASP) activation, receptor activator of nuclear factor-κB ligand (RANKL)/osteoprotegerin (OPG) imbalance, Wnt–sclerostin–Dickkopf-1 (DKK-1) balance, oxidative stress and ferroptosis, and chronic immune activation. These nodes act on a common core of bone-homeostasis disruption — osteoclast activation, osteoblast suppression and osteocyte injury — yet generate distinct skeletal outputs: systemic bone loss in osteoporosis (OP); cartilage degeneration with subchondral remodeling in osteoarthritis (OA); erosion with impaired repair in rheumatoid arthritis (RA); erosion combined with ectopic bone formation in psoriatic arthritis (PsA); and focal high-turnover remodeling in Paget’s disease of bone (PDB). RA and PsA are shown as separate outputs because the direction of Wnt regulation differs between them: DKK-1 and sclerostin are elevated in OP and RA and are associated with suppressed formation and impaired repair, whereas relative DKK-1 reduction in PsA is thought to permit Wnt-dependent ectopic bone formation. The Wnt node is therefore drawn as two branches of opposite sign rather than as a single shared inhibitory step; sign, not membership, distinguishes the two diseases. In OA the same node is engaged with the sign inverted relative to the upper branch, endogenous sclerostin and DKK-1 appearing protective rather than pathogenic; OA is therefore annotated separately rather than assigned to either branch. The five disorders are grouped by etiology rather than by age (Section 1.1): OP and OA are aging-driven, RA and PsA are immune-mediated with aging acting as a modifier, and PDB is included as a boundary case. DKK-1, Dickkopf-1; IL, interleukin; OA, osteoarthritis; OP, osteoporosis; OPG, osteoprotegerin; PDB, Paget’s disease of bone; PsA, psoriatic arthritis; RA, rheumatoid arthritis; RANKL, receptor activator of nuclear factor-κB ligand; SASP, senescence-associated secretory phenotype; TNF-α, tumor necrosis factor-α.

### 1.1 Scope and disease selection

Diseases were selected against three stated criteria: (i) the disorder must have a defined skeletal remodeling phenotype attributable to a shift in osteoclast–osteoblast balance, rather than to primary structural or neoplastic disease; (ii) human evidence must exist for at least three of the five mechanistic nodes examined here; and (iii) the disorder must contribute a distinct skeletal output, so that convergence and divergence can be separated. Five disorders meet these criteria and are analyzed throughout: OP and OA as aging-driven remodeling disorders, in which chronological aging, senescence and low-grade inflammation are primary; RA and PsA as immune-mediated arthritides, in which adaptive immune activation is primary and aging modifies severity, repair capacity and comorbid bone loss; and PDB as a boundary case, in which a largely cell-intrinsic, monogenic mechanism produces a comparable terminal phenotype (Section 6.4). Disorders excluded under these criteria include ankylosing spondylitis (AS), axial spondyloarthritis, osteomalacia, renal osteodystrophy, glucocorticoid-induced osteoporosis and metastatic bone disease. AS is not analyzed as a target disease and appears only in Section 7.4.1, where anti-cytokine trials in AS provide therapeutic precedent for cytokine-directed effects on bone; it is not included in **Figure 3** or **Table 1**. Where evidence for a node in a given disease is absent, the corresponding cell is graded E4 and retained rather than removed, so that gaps remain visible.

**Figure 3:**
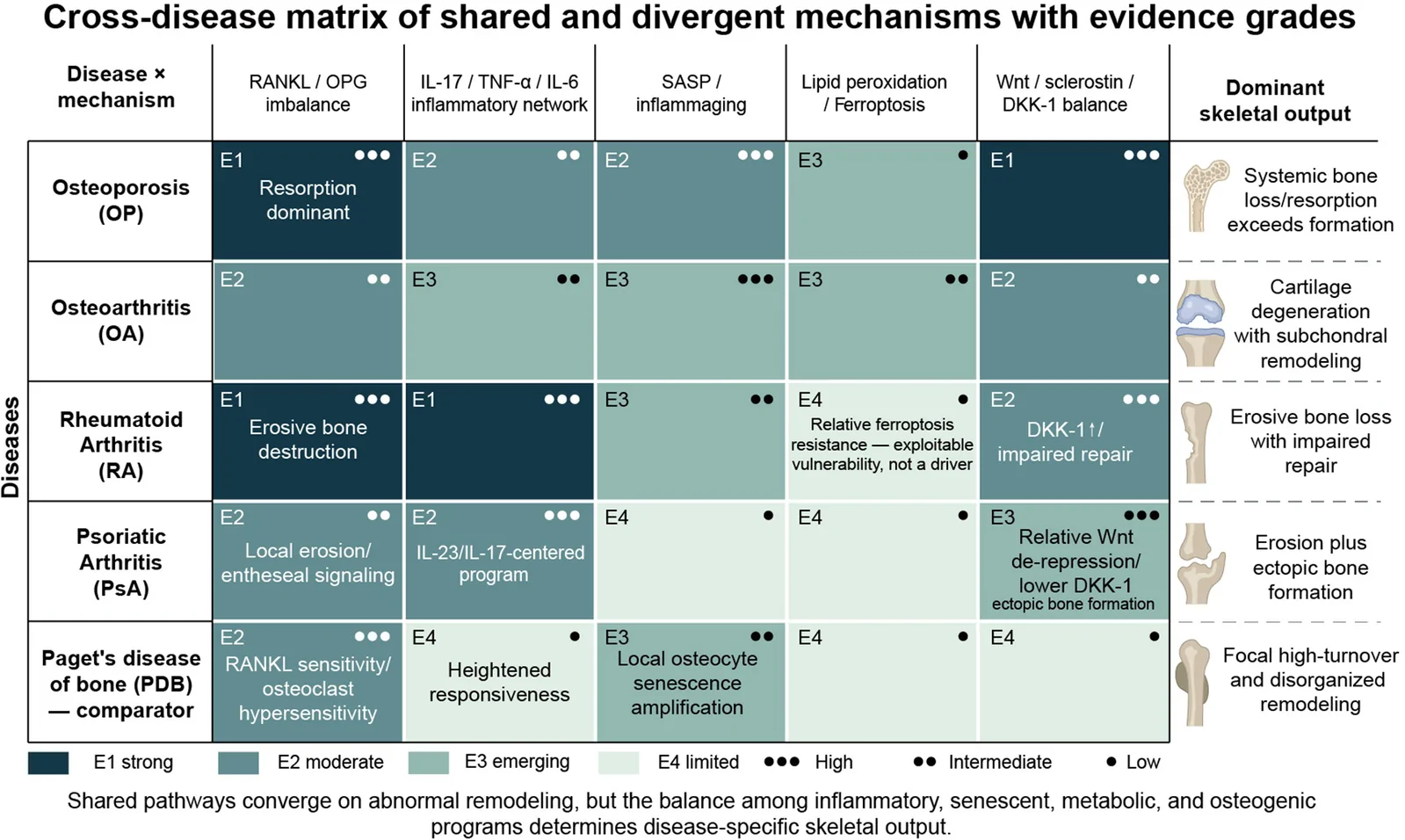
Cross-disease matrix of shared and divergent mechanisms with evidence grades. The matrix compares five shared mechanistic nodes — RANKL/OPG imbalance, the IL-17/TNF-α/IL-6 inflammatory network, SASP/inflammaging, lipid peroxidation and ferroptosis, and Wnt–sclerostin–DKK-1 balance — across OP, OA, RA, PsA and PDB, the last of which is labeled as a comparator rather than as a fifth immune-metabolic disease (Section 6.4). Two properties are encoded separately, because evidence maturity and mechanistic prominence are distinct. Shading encodes the level of evidence supporting each disease × mechanism combination, using the four-level scheme defined *a priori* in Section 2.2: E1/strong (randomized or consistent prospective human evidence with concordant mechanistic support), E2/moderate (reproducible human observational or tissue evidence together with *in vivo* perturbation), E3/emerging (predominantly animal *in vivo* or primary human cell evidence without human outcome data), and E4/limited (*in vitro* data only, single unreplicated reports, or indirect inference). The symbol in the upper-right corner of each cell encodes mechanistic prominence within that disease (••• high, •• intermediate, • low). Grades were assigned independently by two authors on the basis of the highest available evidence tier and adjudicated by the third; cell-level justifications and supporting references are given in **Supplementary Table 1**. Grading is ordinal, not quantitative: cells of the same grade in different diseases are not comparable as effect sizes, and no numerical weighting, citation counting or Delphi procedure was used. Cells for which no disease-specific evidence was retrieved are graded E4 and retained rather than removed, so that gaps in the field remain visible. **Table 1** carries the same grades. DKK-1, Dickkopf-1; IL, interleukin; OA, osteoarthritis; OP, osteoporosis; OPG, osteoprotegerin; PDB, Paget’s disease of bone; PsA, psoriatic arthritis; RA, rheumatoid arthritis; RANKL, receptor activator of nuclear factor-κB ligand; SASP, senescence-associated secretory phenotype; TNF-α, tumor necrosis factor-α.

**Table 1:** Cross-disease comparison of shared pathological mechanisms.

Mechanistic node	OP	OA	RA	PsA	PDB (comparator)
**RANKL/OPG axis**	**E1 · High** Systemic resorption with a reduced OPG/RANKL ratio; RANKL blockade reduces fracture risk.	**E2 · Intermediate** Subchondral remodeling and osteophyte-related turnover; RANKL blockade improves structure in erosive hand OA.	**E1 · High** Synovial RANKL expression with marginal erosion; a RANKL-independent osteoclast route is also described.	**E2 · Intermediate** Entheseal RANKL signaling, with erosion occurring alongside repair.	**E2 · High** Focal osteoclast hypersensitivity generating high-turnover lesions.
**IL-17/TNF-α/IL-6 network** *(includes Th17/Treg balance)*	**E2 · Intermediate** Amplifies osteoclastogenesis after estrogen loss; Th17 expansion with reduced Treg restraint.	**E3 · Intermediate** Macrophage-derived TNF-α, IL-6 and IL-1β promote synovitis; Th17/Treg evidence still emerging.	**E1 · High** Core inflammatory network driving erosive bone loss; established adaptive immune driver.	**E2 · High** IL-23/IL-17-centered program dominates and is central to Th17 biology.	**E4 · Low** Heightened osteoclast responsiveness to inflammatory stimuli; limited direct human evidence.
**SASP/inflammaging**	**E2 · High** Osteocyte, BMSC and immune-cell senescence contribute to bone loss; the phase 2 senolytic trial did not meet its primary endpoint.	**E3 · High** Chondrocyte and synovial senescence amplify degeneration; senescent-cell clearance benefits experimental OA.	**E3 · Intermediate** Secondary amplifier in chronic inflammation; senescent T cells associated with early bone loss.	**E4 · Low** Possible local amplifier; no PsA-specific evidence retrieved.	**E3 · Intermediate** Local osteocyte senescence induced by osteoclast-derived IGF1 may amplify lesion activity.
**Lipid peroxidation/ferroptosis**	**E3 · Low** Osteoblast and osteocyte injury with impaired formation; rodent *in vivo* evidence only.	**E3 · Intermediate** Chondrocyte ferroptosis and cartilage damage through ferritinophagy and TfR1-dependent iron handling.	**E4 · Low** Synovial fibroblast ferroptosis resistance is an exploitable vulnerability rather than a driver of erosion.	**E4 · Low** No PsA-specific evidence retrieved.	**E4 · Low** No PDB-specific evidence retrieved.
**Wnt–sclerostin–DKK-1 balance**	**E1 · High** Suppressed formation; sclerostin inhibition reduces vertebral fracture risk in phase 3 trials.	**E2 · Intermediate** Sclerostin is focally increased in damaged cartilage but reduced in sclerotic subchondral bone, and both sclerostin and DKK-1 are expressed in subchondral osteocytes at the cartilage-damage front. The sign is inverted relative to OP and RA: endogenous Wnt inhibition appears protective, which limits transfer of anti-sclerostin therapy from OP to OA.	**E2 · High** Elevated DKK-1 linked to impaired repair and radiographic progression.	**E3 · High** Relative Wnt de-repression with lower DKK-1 may permit ectopic bone formation.	**E4 · Low** No PDB-specific evidence retrieved.

Qualitative comparison of the dominant or better-supported immune, senescence-related, lipid-metabolic and osteogenic mechanisms across the five selected bone and joint disorders. The disorders are grouped by etiology rather than by age (Section 1.1): OP and OA are aging-driven, RA and PsA are immune-mediated with aging acting as a modifier, and PDB is included as a comparator (Section 6.4). Values in bold within each disease column denote the evidence grade and mechanistic prominence assigned to that disease × mechanism combination, given in the form E · prominence; the plain text beneath each bold value summarizes the supporting findings. Evidence grades were defined a priori by study design (Section 2.2): E1 (strong), randomized or consistent prospective human evidence with concordant mechanistic support; E2 (moderate), reproducible human observational or tissue evidence together with *in vivo* perturbation; E3 (emerging), predominantly animal *in vivo* or primary human cell evidence without human outcome data; E4 (limited), *in vitro* data only, single unreplicated reports, or indirect inference. Prominence descriptors (High, Intermediate, Low) indicate how dominant the mechanistic node is within that disease and correspond to ●●●, ●● and ● in **Figure 4**. Cells for which no disease-specific evidence was retrieved are graded E4 and retained so that evidence gaps remain visible. Bold text in the header row and first column identifies the disease and mechanistic node, respectively, and carries no grading meaning. Evidence grade and prominence are reported separately because evidence maturity and mechanistic prominence are distinct properties. Grades were assigned independently by two authors and adjudicated by the third, and cell-level justifications with supporting references are listed in **Supplementary Table S1**. Grades are ordinal descriptors of study design, not effect sizes or quantitative rankings; no numerical weighting, citation counting or Delphi procedure was used. The same grades are shown in **Figure 4**. BMSC, bone marrow mesenchymal stromal cell; DKK-1, Dickkopf-1; IGF1, insulin-like growth factor 1; IL, interleukin; OA, osteoarthritis; OP, osteoporosis; OPG, osteoprotegerin; PDB, Paget's disease of bone; PsA, psoriatic arthritis; RA, rheumatoid arthritis; RANKL, receptor activator of nuclear factor-κB ligand; SASP, senescence-associated secretory phenotype; TfR1, transferrin receptor 1; TNF-α, tumor necrosis factor-α; Treg, regulatory T cell; Wnt, wingless/integrated signaling pathway.

**Figure 4:**
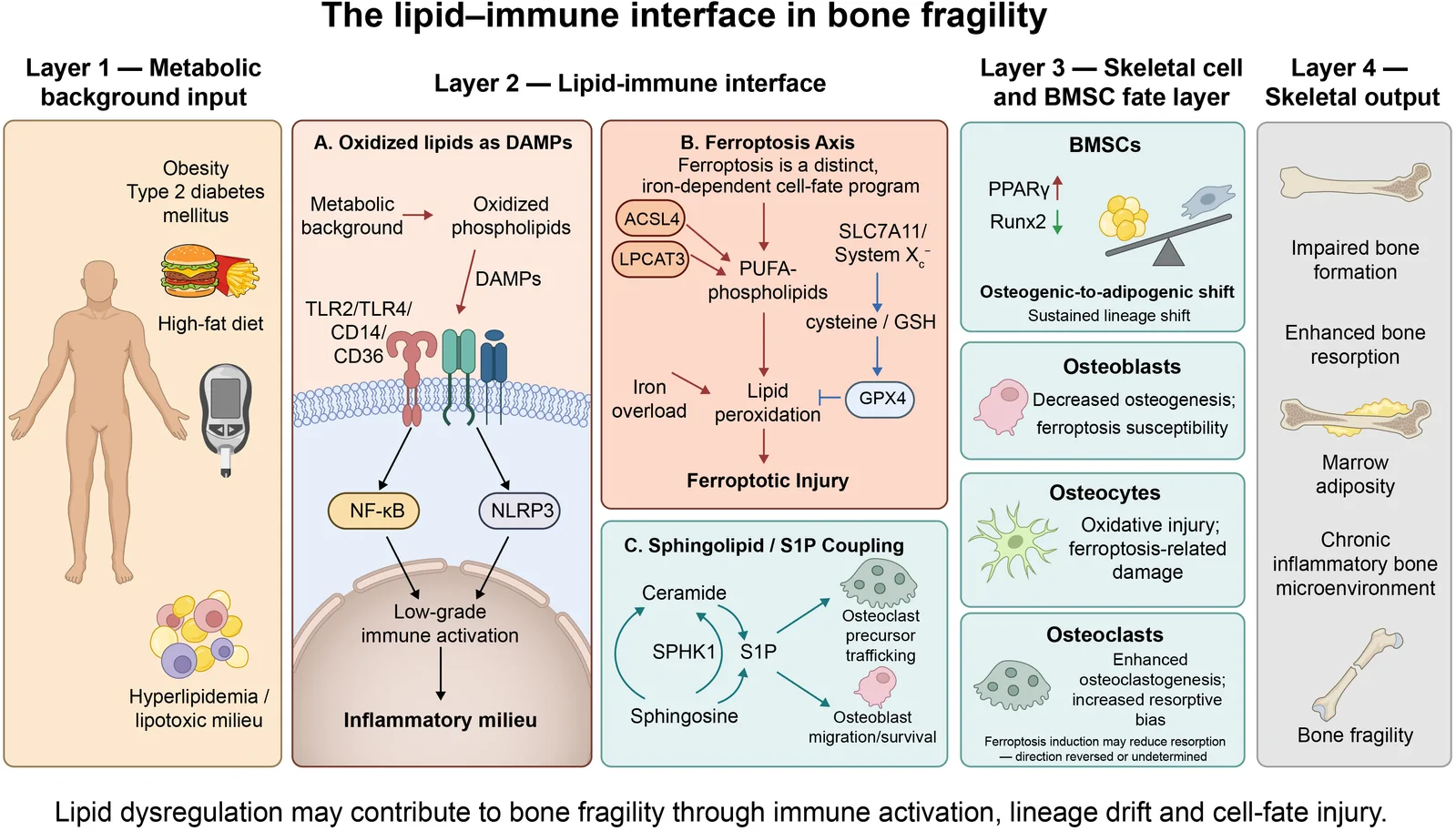
The lipid–immune interface in bone fragility. Metabolic background inputs, including obesity, type 2 diabetes mellitus, high-fat diet, hyperlipidemia, and a lipotoxic milieu, promote oxidized phospholipid accumulation and immune activation. Oxidized lipids act as DAMPs and engage pattern-recognition and scavenger-receptor pathways, including TLR2/TLR4, CD14, and CD36, thereby activating NF-κB and NLRP3-dependent inflammatory signaling. In parallel, PUFA-phospholipid metabolism, ACSL4/LPCAT3 activity, iron overload, GPX4 dysfunction, and SLC7A11/system Xc− impairment increase lipid peroxidation and ferroptotic injury. Sphingolipid and S1P signaling further couple lipid metabolism to osteoclast precursor trafficking and osteoblast survival. Together, these processes drive BMSC adipogenic shift, impaired osteoblastogenesis, osteocyte oxidative injury, enhanced osteoclastogenesis, marrow adiposity, chronic inflammatory remodeling, and bone fragility. The inhibitory arm of the ferroptosis axis is drawn as SLC7A11/system Xc^−^ → cysteine/glutathione → GPX4 ⊣ lipid peroxidation; ACSL4 and LPCAT3 are shown only as promoters of polyunsaturated fatty acid (PUFA)-phospholipid synthesis. ACSL4, acyl-CoA synthetase long-chain family member 4; BMSC, bone marrow stromal/stem cell; DAMPs, damage-associated molecular patterns; GPX4, glutathione peroxidase 4; LPCAT3, lysophosphatidylcholine acyltransferase 3; NF-κB, nuclear factor-κB; NLRP3, NOD-like receptor family pyrin domain-containing 3; PPARγ, peroxisome proliferator-activated receptor-γ; PUFA, polyunsaturated fatty acid; Runx2, runt-related transcription factor 2; S1P, sphingosine-1-phosphate; SLC7A11, solute carrier family 7 member 11; SPHK1, sphingosine kinase 1; TLR, Toll-like receptor.

### 1.2 Review question and conceptual advance

Osteoimmunology, inflammaging/senescence biology and lipid-metabolic bone research have each been reviewed thoroughly, and we do not claim to have identified new mechanisms. This Review addresses one question that the constituent literatures do not answer separately: do these three programs act on a shared, enumerable set of regulatory nodes, and if so, what determines why the same nodes yield systemic bone loss in one disease, cartilage failure with subchondral remodeling in another, and erosion combined with ectopic bone formation in a third? Our contribution consists of four specific claims. First, we specify the convergence set instead of asserting convergence in general terms: five nodes — RANKL/OPG, the interleukin (IL)-17/TNF-α/IL-6 network, senescence-associated secretory phenotype (SASP)/inflammaging, phospholipid peroxidation and ferroptosis, and Wnt–sclerostin–DKK-1 balance — are proposed as sufficient to describe the terminal uncoupling step in all five disorders. Second, we propose that disease identity is determined by the sign and relative weighting of node activity rather than by which nodes are engaged; the opposite direction of DKK-1 regulation in RA and PsA is the clearest worked example (17, 18), and it predicts skeletal output better than inflammatory intensity does. Third, we bring the lipid/ferroptosis axis, which is usually reviewed within metabolic bone disease and rarely inside osteoimmunology, into the same node set, and treat its direction as cell-type-resolved rather than tissue-level (Section 5.2). Fourth, we make the framework auditable and testable by grading every disease × mechanism cell (Section 2.2), with the full graded matrix reported in **Figure 3** and **Table 1**, so that weakly supported cells become explicit research targets rather than implicit assertions.

The framework yields three falsifiable predictions. (i) Senolytic efficacy in OP is conditional on baseline senescent-cell burden rather than uniform across patients — partially supported by the subgroup response in the phase 2 D+Q trial (16), and refutable by a burden-stratified trial showing no interaction. (ii) The direction of DKK-1 change, not its absolute concentration, predicts whether inflammatory arthritis produces erosive or ectopic skeletal output; a cohort in which high-DKK-1 PsA still develops enthesophytes would refute this. (iii) Unselective ferroptosis-directed therapy will produce offsetting effects on formation and resorption, so net skeletal benefit requires cell-selective delivery; demonstration of a net gain in bone mineral density (BMD) with a systemic, non-targeted glutathione peroxidase 4 (GPX4) activator would refute it. Each prediction names the design that would falsify it, which is the sense in which we intend the framework to be more than a summary.

## 2 Review approach and evidence grading

### 2.1 Literature search and selection

This is a narrative, mechanism-oriented review. It is not registered as a systematic review, does not claim exhaustive coverage, and no PRISMA flow diagram is presented; the search was nonetheless structured so that the evidence base is auditable. We searched PubMed/MEDLINE, Embase and the Web of Science Core Collection from 1 January 2000 to 31 July 2026, with ClinicalTrials.gov for interventional evidence, combining four concept blocks with Boolean operators: the five disorders; the five mechanistic nodes of the framework; interventions directed at those nodes; and biomarker and skeletal outcome terms. MeSH and Emtree descriptors were used where a controlled-vocabulary term exists and title/abstract free text otherwise. Reference lists and forward citations of key mechanistic papers were screened manually, and current EULAR and ASAS–EULAR recommendations and Global Burden of Disease estimates were retrieved directly. Titles and abstracts were screened by two authors independently, full texts were assessed in duplicate, and disagreements were resolved with the third author. The complete term list, the inclusion and exclusion criteria and the screening procedure are given in **Supplementary Methods S1**.

Retrieval was iterative and purposive rather than a single pre-specified query, so no record counts are reported and selection bias cannot be excluded; we mitigate this by grading every disease × mechanism claim (Section 2.2) and by retaining and flagging weakly supported entries rather than omitting them. The review cites 160 references, of which 89% appeared in 2020 or later. The search window opens on 1 January 2000, but the retained citation set contains no publication from 2000 to 2010. This follows from the selection rule rather than from an additional date filter: where a mechanism was first described before 2011, the current authoritative account is cited in place of the original report, and two of the five nodes have little pre-2011 primary literature to cite, ferroptosis having been defined in 2012 and the SASP/inflammaging framework consolidated after 2010. The retained references span 2011 to 2026.

### 2.2 Evidence grading

Each disease × mechanism combination was assigned *a priori* by study design rather than by perceived importance. E1 (strong): randomized trial with skeletal or structural endpoints, or consistent large prospective human cohorts, with concordant mechanistic support. E2 (moderate): reproducible human observational or human tissue evidence together with *in vivo* genetic or pharmacological perturbation in at least one animal model. E3 (emerging): predominantly animal *in vivo* or primary human cell evidence, with limited replication and no human outcome data. E4 (limited or indirect): *in vitro* data only, single unreplicated reports, or inference from a non-skeletal tissue or another disease. Mechanistic prominence within a disease (high, intermediate, low) is graded separately, because evidence maturity and prominence are distinct properties. Two authors graded independently and the third adjudicated. The same four levels are applied inline in Sections 3 to 8 to individual statements. Grades are ordinal descriptors of study design and not effect sizes, and cells for which no disease-specific evidence was retrieved are graded E4 and retained so that gaps in the field remain visible. Cell-level justifications and supporting references are given in **Supplementary Table 1**.

## 3 Foundations of osteoimmunology: the immune–skeletal regulatory network

This section sets out the cellular and molecular basis of the shared nodes specified in Section 1.2 — the RANKL/OPG axis, the IL-17/TNF-α/IL-6 network, and SASP/inflammaging — and is the single location in which the RANKL–RANK–OPG circuitry is developed in full; Sections 6 and 7 refer back to it and discuss only the differences between diseases rather than restating the mechanism. The remaining two nodes, phospholipid peroxidation and ferroptosis and Wnt–sclerostin–DKK-1 balance, are developed in Section 5. Grades in parentheses follow the scheme defined in Section 2.2.

### 3.1 Cellular basis of bone remodeling

Mature bone tissue is maintained by osteoblasts, osteoclasts, osteocytes, and bone marrow mesenchymal stromal cells (BMSCs). BMSC fate decisions sit upstream of bone homeostasis. Under coordinated input from Wnt, BMP, TGF-β, Hedgehog, and PTH signals, BMSCs commit to the osteoblast lineage through transcription factors including Runx2, Osterix, and ATF4 (19). Runx2 expression in the osteoblast lineage varies in a stage-dependent manner and drives induction of bone matrix protein genes such as *Col1a1, Spp1, Ibsp* and *Bglap* (20). The adipogenic–osteogenic fate of BMSCs also responds to chemical, mechanical, and metabolic cues; PPARγ activation favors adipogenesis, while elevated Runx2 favors osteogenesis (21).

Osteoclasts arise from monocyte–macrophage precursors and fuse into mature multinucleated cells in the presence of macrophage colony-stimulating factor (M-CSF) and RANKL (22). Earlier work treated osteoclasts as terminally differentiated cells that undergo apoptosis once resorption is complete. Intravital imaging shows that some osteoclasts undergo fission into transcriptionally distinct daughter cells called osteomorphs, which can re-fuse to form new osteoclasts (23) (E3: intravital imaging and transcriptomics in mice, without human confirmation). This finding offers a partial cellular explanation for the rebound resorption observed after denosumab withdrawal (24), a clinical phenomenon that is itself well documented (E1) even though the osteomorph account of it is not (E3).

Osteocytes account for roughly 90–95% of bone cells and serve as mechanosensors and endocrine hubs (25). Under reduced mechanical loading, osteocytes express sclerostin, which binds LRP5/6 to antagonise Wnt signaling and suppress bone formation; loading regulates the system in the opposite direction (25). Osteocytes also secrete RANKL and OPG and function as the coupling node between formation and resorption (25).

### 3.2 The RANKL–RANK–OPG Axis

RANKL (TNFSF11), RANK (TNFRSF11A), and OPG (TNFRSF11B) form the core circuit of bone remodeling. RANKL produced by osteoblasts, osteocytes, and activated T cells binds RANK on osteoclast precursors, recruits TRAF6, and activates NF-κB, MAPK, and NFATc1 to drive osteoclast differentiation (26). OPG serves as a soluble decoy receptor that blocks RANKL–RANK engagement, and the RANKL/OPG ratio determines the pace of resorption (26). A meta-analysis of five randomized controlled trials found lower serum OPG/RANKL ratios in OP patients than in controls, though the association of OPG or RANKL alone with OP varies across studies (27) (E2: reproducible human observational evidence with assay heterogeneity).

RANKL was first identified in immunology, where it participates in T cell–dendritic cell interactions and lymphoid organogenesis, placing the axis naturally within osteoimmunology (28). Under pathological conditions, activated T cells, B cells, and tumor cells can act as RANKL sources. In ovariectomy models, B cell-specific *Hif1a* deletion reduces susceptibility to estrogen-deficient bone loss; estrogen itself promotes HSP70-mediated degradation of HIF-1α in B cells and limits B-cell RANKL output (29) (E3: genetic perturbation in mice only). On the therapeutic side, the fully human anti-RANKL antibody denosumab has become a mainstay antiresorptive agent for postmenopausal OP (30) (E1: randomized fracture-endpoint evidence).

This axis is the most strongly supported of the five nodes and the only one for which a licensed, node-directed therapy exists. It is also the node at which the five disorders differ least in mechanism and most in setting: systemic in OP, local and pannus-associated in RA and PsA, and osteoclast-intrinsic in PDB. Those differences are treated in Sections 6.1 and 6.3 and are not restated here.

### 3.3 Innate immunity and bone homeostasis

Innate immune cells share the bone marrow niche with bone cells, and several populations participate directly in remodeling. Macrophages and osteoclasts arise from the same monocyte–macrophage precursors. M1 polarization is characterized by secretion of TNF-α, IL-1β, and IL-6, which upregulate RANKL and promote resorption. M2 polarization secretes IL-10, TGF-β, BMP-2, and VEGF, supporting bone formation and the vascular–skeletal interface (31). An elevated M1/M2 ratio in the OP bone marrow microenvironment is associated with reduced bone density (31) (E3).

Under chronic inflammation and aging, neutrophil chemotaxis, transmigration, and apoptosis become impaired. The cells secrete IL-23, G-CSF, and reactive oxygen species, amplifying IL-17 signaling and promoting osteoclastogenesis (32): the contribution of neutrophils is therefore indirect, exerted through the IL-23/IL-17 milieu rather than through a direct action on osteoclast precursors (E3), and **Figure 1** has been redrawn to state this explicitly. The peripheral blood neutrophil-to-lymphocyte ratio (NLR) is associated with low bone density in postmenopausal women and may serve as an auxiliary marker of systemic inflammatory burden (33) (E2 as an association; the causal direction is untested). The role of NK cells is context-dependent. In inflammatory joint diseases such as RA and PsA, joint-infiltrating NK cells express RANKL and M-CSF, cooperating with monocytes to induce osteoclastogenesis. IFN-γ secreted by NK cells can also inhibit osteoclast differentiation, and the temporal relationship between these opposing effects remains incompletely defined (34) (E3; the two effects have not been separated in the same human tissue).

Osteoclast differentiation depends on RANK signaling together with co-stimulation through immunoreceptor tyrosine-based activation motif (ITAM) adaptors. DAP12 and FcRγ couple to immunoglobulin-like receptors such as OSCAR and TREM-2, activate PLCγ2 and Ca^2+^ signaling, and amplify NFATc1 nuclear translocation and expression (26). This pathway links innate immune recognition with osteoclast differentiation.

### 3.4 Adaptive immunity and bone remodeling

The balance among CD4^+^ T cell subsets sits at the center of adaptive immune regulation of bone remodeling. Th17 and Treg cells share a common CD4^+^ T precursor, with TGF-β, IL-6, and IL-23 directing differentiation. Th17 cells secrete IL-17 and express RANKL, promoting osteoclastogenesis. Tregs secrete IL-10 and TGF-β, express CTLA-4, and inhibit osteoclast differentiation while supporting bone formation (35). In RA and postmenopausal osteoporosis (PMOP), a rising Th17/Treg ratio parallels enhanced resorption (35) (E2).

Aging is accompanied by a higher peripheral Th17 fraction and reduced Treg function, consistent with the low-grade chronic inflammation that defines inflammaging (36). In inflammaging-related animal models, IL-17 and IFN-γ act together to enhance RANKL-driven osteoclastogenesis and alveolar bone destruction, suggesting that the cytokine combination found in older individuals has stronger resorptive potential (37) (E3: animal models of periodontal bone loss, not yet reproduced in human skeletal endpoints).

B cells in the resting state are an important source of bone marrow OPG. Under estrogen deficiency, HIF-1α signaling in B cells remains active and RANKL output increases. Mice with B cell-specific *Hif1a* deletion are protected from ovariectomy-induced bone loss (29). This work establishes B cells as an independent regulatory node in the bone–immune network.

### 3.5 Cytokine networks and immune aging

Bone–immune homeostasis is maintained by a set of interconnected cytokines. TNF-α is secreted by M1 macrophages, activated T cells, and synovial fibroblasts. It upregulates RANKL and RANK through NF-κB and MAPK and directly promotes osteoclast differentiation (38). IL-1β upregulates RANKL and enhances its activity, with positive feedback between IL-1β and TNF-α (38).

IL-6 signals through two routes, classical (cis) and trans. At low RANKL concentrations, IL-6/sIL-6R amplifies osteoclastogenesis through NF-κB and JNK; sgp130-mediated inhibition of trans-signaling attenuates this effect (39). In RA synovial fibroblasts, IL-6 trans-signaling induces osteoclast marker genes such as *CTSK* and *CTSB* via the transcription factor Ets-2 and promotes formation of bone-like resorption pits (40) (E3: human cell evidence without *in vivo* confirmation). IL-17 directly upregulates RANKL in osteoblasts and synovial fibroblasts and induces expression of TNF-α, IL-1, and IL-6, forming a positive feedback loop with RANKL (38). IL-10, TGF-β, and IL-4 produced by Tregs, M2 macrophages, and Th2 cells downregulate RANKL and upregulate OPG. Bone repair typically shows temporal switching between M1–M2 and Th17–Treg phases, and mistimed transitions may impair healing (31).

The SASP links the cytokine network to the aging–immune–bone axis. In the senescent bone marrow microenvironment, aged macrophages and neutrophils secrete GCA, which binds plexin-B2 on BMSCs, suppresses osteogenesis, and promotes adipogenic differentiation (11) (E3: rodent models with human tissue correlation only). SASP-related IL-6, IL-8, and CCL2 also affect the RANKL/OPG ratio and negatively influence bone remodeling in older adults (11). This is the single full account of the grancalcin–plexin-B2 axis in the Review; Section 4.4 refers to it rather than repeating it.

Osteoimmunology moves bone remodeling beyond a purely mechanical–metabolic frame and into an immune-regulatory one, while offering a candidate list of therapeutic targets shared across diseases. Denosumab, anti-sclerostin antibodies, anti-IL-6 and anti-IL-17 monoclonal antibodies, and strategies aimed at the Th17/Treg balance or at senescent cells have become the main directions of bone–immune target research. Clinical translation around subtype identification, long-term safety, and post-withdrawal rebound still requires more prospective evidence. Two points from this section carry forward into the framework. The cytokine node is engaged in all five disorders, so it does not by itself discriminate between them; and the same node can act in opposite directions depending on the cellular source and the timing of the M1–M2 and Th17–Treg transitions. It is the sign and weighting of node activity, not its presence, that separates the diseases (Section 1.2; **Figure 3**).

## 4 Skeletal aging and immunosenescence as upstream initiators

Bone fragility is not explained by calcium-phosphate metabolism and estrogen withdrawal alone. Over the past decade, cellular senescence, chronic low-grade inflammation and immune-system aging have been folded into a single upstream pathway, often described as a skeletal-aging to immunosenescence to bone-fragility continuum (41). This section follows that sequence, from molecular to systemic, and corresponds to the SASP/inflammaging node of the framework; the layered version of the same sequence is shown in **Figure 5**.

**Figure 5:**
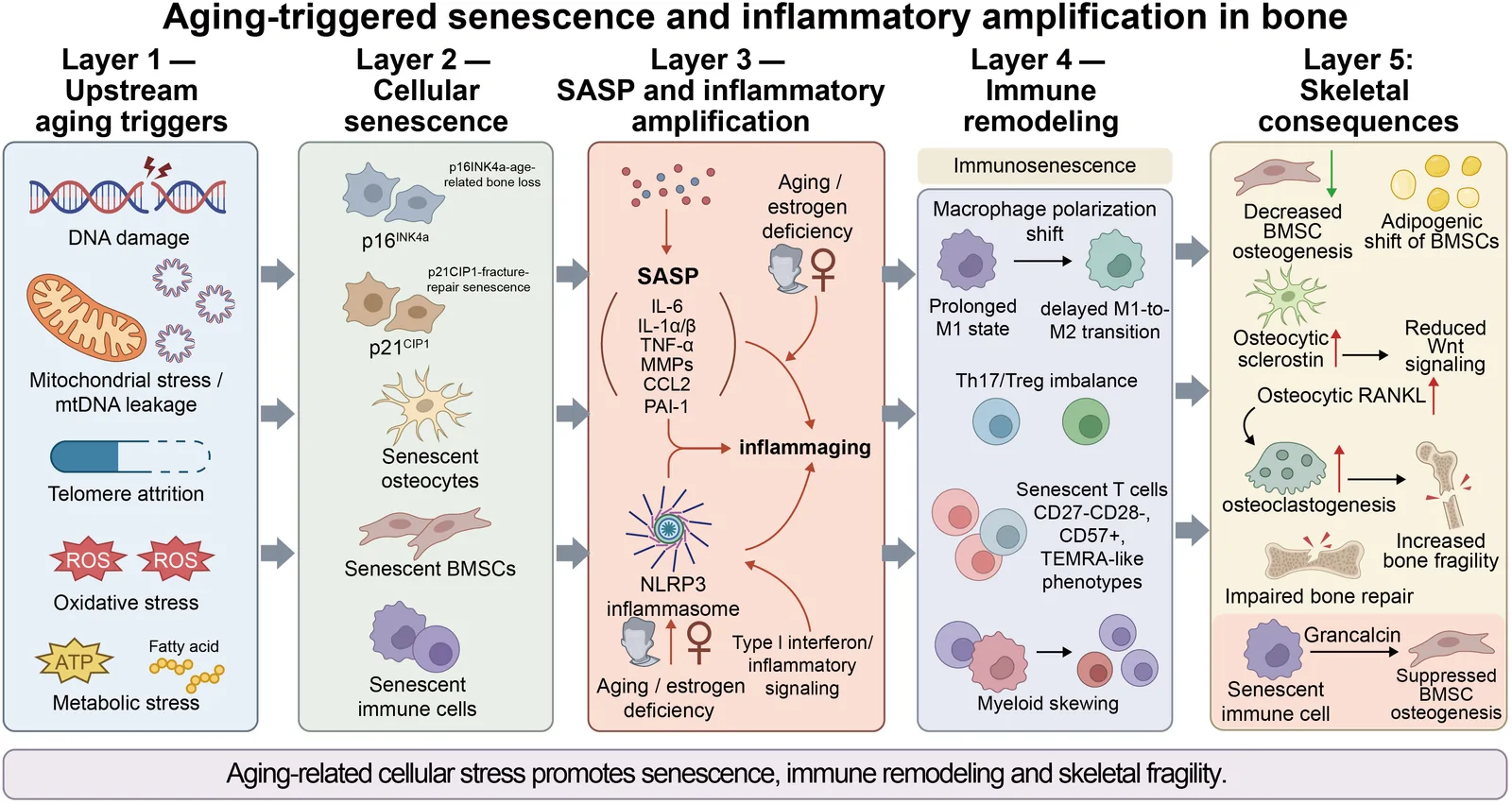
Aging-triggered senescence and inflammatory amplification in bone. Upstream aging insults, including DNA damage, mitochondrial stress, mtDNA leakage, telomere attrition, oxidative stress, and metabolic stress, induce senescence programs in osteocytes, BMSCs, and immune cells. Senescent cells secrete SASP factors, activate the NLRP3 inflammasome, and reinforce inflammaging, particularly in the setting of aging and estrogen deficiency. These signals remodel immune function through macrophage polarization, Th17/Treg imbalance, senescent T-cell accumulation, and myeloid skewing. The downstream skeletal consequences include reduced BMSC osteogenesis, adipogenic lineage shift, osteocytic RANKL upregulation, increased osteoclastogenesis, impaired Wnt signaling, defective repair, and bone fragility. In this figure, p16 INK4a -positive cells are labeled as the population associated with age-related bone loss and p21 CIP1-positive cells as the population associated with fracture-repair senescence. ATP, adenosine triphosphate; BMSC, bone marrow stromal/stem cell; CCL2, C-C motif chemokine ligand 2; CD, cluster of differentiation; IL, interleukin; MMPs, matrix metalloproteinases; mtDNA, mitochondrial DNA; NLRP3, NOD-like receptor family pyrin domain-containing 3; PAI-1, plasminogen activator inhibitor-1; RANKL, receptor activator of nuclear factor-κB ligand; ROS, reactive oxygen species; SASP, senescence-associated secretory phenotype; TEMRA, terminally differentiated effector memory T cells re-expressing CD45RA; TGF-β, transforming growth factor-β; Th17, T helper 17 cell; TNF-α, tumor necrosis factor-α; Treg, regulatory T cell; Wnt, wingless/integrated signaling pathway.

### 4.1 Cellular senescence and the SASP

Cellular senescence is a stable cell-cycle arrest triggered by DNA damage, telomere shortening, oxidative stress or oncogenic signaling. The 2023 update of the hallmarks of aging places cellular senescence and chronic inflammation among the core interlocking features (42). Senescent cells stop dividing and also acquire the SASP, releasing soluble factors that include IL-6, IL-8, IL-1α/β, TNF-α, matrix metalloproteinase (MMP)-3/9, CCL2 and PAI-1 (43). In the short term the SASP recruits immune cells to clear damaged tissue. When sustained, it disturbs the surrounding microenvironment.

In bone, p16^INK4a^-positive senescent cells are found in osteocytes, marrow mesenchymal progenitors and myeloid immune cells (43). A CyTOF-based multiparametric study identified CD24^high^ osteolineage cells as a population that can be eliminated by senolytics (44). (E3). That work moved bone senescence from a single-marker concept toward a multiparameter cellular phenotype.

Metabolic stress accelerates the cellular age of bone tissue. In a mouse model of type 2 diabetes, osteocytes acquire p16 and γH2AX positivity earlier than during normal aging, with an amplified SASP and earlier deterioration of microarchitecture and biomechanical strength (45). (E3). In BMSCs, aging is accompanied by increased oxidative stress, impaired autophagy and an expanded SASP, with reduced osteogenic capacity and a drift toward adipogenesis (46).

A subset of senescent cells is driven mainly by p21^WAF1/CIP1^. After fracture, the callus rapidly contains p21-positive osteochondroprogenitors and a p21-positive neutrophil subset whose SASP differs from the p16-positive senescent cells associated with chronological aging (47). Genetic clearance of p21-positive cells accelerates fracture repair but does not affect age-related bone loss; clearance of p16-positive cells does the opposite (47). At least two relatively independent senescent lineages coexist in bone. The two markers therefore map onto different skeletal outcomes — p16^INK4a^ to age-related bone loss and p21^WAF1/CIP1^ to fracture-repair senescence (E3) — and **Figure 5** carries the same assignment. This distinction also predicts that a senolytic selective for one lineage should not be expected to act on the other.

### 4.2 Inflammaging in the bone microenvironment

Inflammaging refers to systemic, low-grade, sterile inflammation that increases with age (41). Its molecular sources include the SASP, T-cell redifferentiation driven by persistent viruses, endotoxin translocation from a dysbiotic gut, and accumulation of damage-associated molecular patterns (DAMPs). C-reactive protein (CRP), IL-6 and TNF-α are higher in older adults than in younger controls, and these elevations correlate with all-cause mortality (41).

Bone is one of the main target tissues. In older individuals, macrophages at the fracture site show heightened sensitivity to inflammatory signals and a delayed M1 to M2 transition, leading to a prolonged acute inflammatory phase, increased osteoclast activity and suppressed osteoblast function (48). This pattern has been independently described in studies of fracture repair (49) (E3, reproduced across two independent model systems).

The NOD-like receptor family pyrin domain-containing 3 (NLRP3) inflammasome links inflammaging to bone remodeling: estrogen loss and aging together drive NLRP3 overactivation, which releases mature IL-1β and IL-18 through caspase-1 and triggers pyroptosis in macrophages, endothelial cells, BMSCs and osteoblasts (50), and the FoxO1/NLRP3 axis shows age-dependent activation in an alveolar bone model (51) (E2–E3). Because NLRP3-driven pyroptosis is one of several regulated cell-death programs acting on the same targets, it is compared with apoptosis, ferroptosis and necroptosis in Section 5.2.1 and is not developed further here.

The cGAS-STING pathway connects DNA damage to inflammaging. Cytosolic dsDNA arising from fragmented nuclear DNA, mitochondrial DNA leakage and reactivated retroelements is recognized by cGAS, which signals through STING, TBK1 and IRF3 to drive type I interferon and SASP-related transcription (52). In stromal cells, declining YAP/TAZ mechanotransduction releases cGAS-STING from inhibition and accelerates aging (53) (E3).

The gut microbiota acts as one of the mediators carrying inflammaging toward bone. Patients with osteoporosis show altered fecal microbial composition, and several genera are inversely associated with BMD (54) (E2 as a human association; E4 for the causal step, which has not been demonstrated in humans). Short-chain fatty acids, particularly butyrate, support Treg differentiation, restrain osteoclasts and maintain the gut barrier (55). Dysbiosis reduces short-chain fatty acid supply, weakens the gut barrier and increases circulating lipopolysaccharide (LPS), which raises systemic inflammatory tone.

Bone marrow adipose tissue (BMAT) provides local amplification. Chemical-shift water-fat MRI across multiple skeletal sites shows that marrow fat content rises with age and is particularly high in the vertebrae of postmenopausal women (56) (E2: consistent human imaging evidence). BMAT secretes adipokines and pro-inflammatory cytokines, releases free fatty acids through local lipolysis, and supports the accumulation of senescent BMSCs (57). BMAT is where the SASP/inflammaging node meets the lipid node; its metabolic side is developed in Section 5.5.

### 4.3 Immunosenescence and immunoporosis

Immunosenescence is the age-related structural and functional decline of the immune system. It is the counterpart of inflammaging (36). Its features include thymic involution, with reduced naïve T-cell output and contraction of the TCR repertoire, myeloid skewing of hematopoietic stem cells, accumulation of memory T cells and CD45RA-re-expressing terminally differentiated effector memory T cells (TEMRA), and reduced dendritic-cell antigen presentation (36). Thymic involution is the anatomical core of immunosenescence. Thymic stroma deteriorates and epithelial cells are progressively replaced by adipose tissue, with parallel reductions in naïve T-cell output and peripheral TCR diversity (58).

The peripheral T-cell pool is remodeled. Repeated antigen exposure, persistent CMV infection and telomere erosion drive the expansion of CD27^−^CD28^−^, CD57^+^ and TEMRA cells, which acquire innate-like cytotoxic activity and secrete IFN-γ and TNF-α (59). Single-cell analyses identify clonal GZMK^+^ CD8 T cells as a conserved feature of inflammaging (60) (E2 in human blood and tissue; the skeletal consequence of this population is untested, E4).

Myeloid skewing of hematopoietic stem cells is the cellular basis of immunosenescence. Chronic inflammatory exposure produces long-lasting impairment of self-renewal and accelerated aging at the molecular level (61). The pool of M-CSF- and RANKL-responsive cells in the marrow expands. Aged macrophages respond more strongly to inflammatory signals and resolve them less well, with a higher early M1 peak and suppressed M2 induction at the fracture callus (49).

Senescent immune cells lose the ability to clear senescent cells in other tissues. In hematopoietic cell-specific *Ercc1* knockout mice, splenocytes from such animals or from aged wild-type mice induce senescence in young recipients, whereas young splenocytes attenuate it (62) (E3: a single well-controlled model system without independent replication). In that model the aged immune system functions as a causal driver of systemic aging.

In the bone field, the concept of immunoporosis places immune cells at the center of osteoporosis pathogenesis (63). Within this framework, innate and adaptive immune cells, senescent immune cells, Th17/Treg balance and the SASP are integrated into the causal chain that previously emphasized only estrogen loss or vitamin D deficiency (64) (E4 as a construct: immunoporosis organizes existing observations rather than resting on evidence of its own, and we use it here as a descriptive label, not as a demonstrated causal model).

### 4.4 Senescent osteocytes, RANKL and bone-formation–resorption uncoupling

Osteocytes are the longest-lived cells in bone. *Tnfsf11* (RANKL) transcription rises in cortical bone of aged mice in parallel with markers of senescence such as p16, p21 and GATA4. Conditional deletion of *Tnfsf11* in osteocytes protects against age-related cortical bone loss without affecting trabecular bone loss, and senolytic treatment reduces cortical RANKL mRNA *in vivo* (65) (E3). A relatively direct path links senescent osteocytes, elevated RANKL and an increase in cortical osteoclasts, and this is the point at which the senescence node feeds into the RANKL/OPG node described in Section 3.2.

The contribution of skeletal aging can be separated into cell-intrinsic and systemic components. In p16-LOX-ATTAC mice, eliminating senescent osteocytes alone reduces age-related bone loss in the spine but not the femur, while systemic clearance of senescent cells improves BMD at both sites, raises osteoblast activity and lowers osteoclast and marrow adipocyte numbers (66) (E3; the site specificity has no human counterpart).

Clinical evidence comes from a single phase 2 randomized controlled trial, described here in full and cross-referenced elsewhere in the Review rather than restated. Postmenopausal women given intermittent dasatinib plus quercetin (D+Q) for 20 weeks showed no change in the resorption marker β-isomerized C-terminal telopeptide of type I collagen (β-CTX) compared with controls, while the formation marker P1NP increased at weeks 2 and 4. In the subgroup with the highest senescent-cell burden, D+Q produced concurrent decreases in resorption, increases in formation and improvement in distal radius BMD (16). The response in that subgroup suggests that baseline senescent-cell burden may shape the therapeutic effect, but the sample size is small and a larger trial is required. Graded explicitly: the trial is E2 — randomized, but powered on a bone-turnover marker rather than on a structural or fracture endpoint, with a negative primary result — while the subgroup finding is E4, being *post hoc* and unreplicated. The distinction matters because the burden-dependence of senolytic response is the first of the three predictions set out in Section 1.2, and it is currently supported only at E4.

Vascular and skeletal coupling is impaired by aging. Senescent bone-endothelial cells suppress Wnt signaling in BMSCs through paracrine factors, with a decrease in Type H vessels that limits the coupling of osteogenesis and angiogenesis (67) (E3).A representative molecular link between immunosenescence and skeletal aging is the grancalcin–plexin-B2 axis, described in Section 3.5 (11).

### 4.5 Senescence as an upstream amplifier

Senescent immune and stromal cells can propagate local signals into systemic bone fragility. Transplantation of senescent immune cells into young recipients induces multi-organ senescence (62). After fracture, p21-positive neutrophils and osteochondroprogenitors drive surrounding stromal cells into senescence through paracrine signals, amplifying the local injury at a distance (47). This aging–senescence–inflammatory sequence is summarized in **Figure 5**, whose terminal outputs are impaired bone repair and increased bone fragility.

Outcomes after hip fracture in older adults are consistent with this amplifier model. One-year mortality remains in the range of 15%–30% worldwide, and fewer than half of survivors regain their pre-fracture function (68) (E1 for the epidemiology; E4 for its attribution to the senescence amplifier, which is an interpretation rather than a tested link). Within this framework, a hip fracture is understood as a systemic event that reflects, rather than only triggers, systemic vulnerability. Metabolic stress speeds up amplification. In a mouse model of type 2 diabetes, the combination of high-fat diet and glucose dysregulation drives osteocytes into premature senescence with deterioration of microarchitecture and biomechanical strength (45).

Periodontal tissue provides additional evidence. In an inflammaging-related animal model, the combination of IL-17 and IFN-γ amplifies RANKL-driven osteoclastogenesis and accelerates alveolar bone loss (37). The result connects systemic inflammaging to local bone destruction in a testable way.

These conclusions are tempered by the fact that most of the evidence comes from animal models and small clinical studies. Applying the scheme of Section 2.2 to this section as a whole, the senescence-to-fragility sequence rests predominantly on E3 evidence, with E1 available only for the epidemiological endpoints and for antiresorptive efficacy, and with no E1 or E2 support for senolytic modification of skeletal outcome. The causal direction across the skeletal-aging, immunosenescence and bone-fragility continuum is not fully resolved. Senescent phenotypes of myeloid and mesenchymal marrow cells differ, and senolytic responses vary between them; a single senolytic regimen applied uniformly across patients is unlikely to be optimal (16, 44). Biomarker stratification, post-treatment rebound and long-term safety remain open questions.

Skeletal aging and immunosenescence form the upstream initiating segment of bone fragility. The SASP is their shared molecular vocabulary, inflammaging is their systemic phenotype, and elevated RANKL with reduced Wnt signaling are their meeting points on the bone-remodeling axis. Whether this upstream segment can be turned into a clinically usable target for anti-skeletal-aging therapy will depend on the results of multi-center randomized trials.

## 5 Phospholipid and lipid metabolic dysregulation: an overlooked metabolic–immune interface

Bone metabolism has long been interpreted within an endocrine framework centered on calcium, phosphorus, vitamin D, and estrogen. Lipids have often been reduced to either an energy substrate or inert filler in the bone marrow. Recent work places lipids back into the core pathways of bone metabolism. Lipids serve three roles at once: energy source, structural component of membranes, and signaling molecule, and they connect metabolic dysregulation to the innate immune interface through oxidized lipid species (69). The layered lipid–immune interface is illustrated in **Figure 4**.

### 5.1 Three roles of lipids in bone metabolism

Matrix protein secretion and mineral vesicle trafficking in osteoblasts are energy-demanding. Glucose is not the only substrate. A study using conditional knockout mice showed that lipid droplets in osteoblasts release free fatty acids through ATGL-driven lipolysis. These fatty acids enter mitochondrial β-oxidation and help maintain ATP levels (70). Under high-fat diet and type 2 diabetes mellitus (T2DM) conditions, perilipin 2 deletion in osteoprogenitors enhances fatty acid oxidation and mitigates diabetes-related bone fragility (71).

Osteoclasts also depend on lipid metabolism. Bone resorption requires substantial ATP, produced jointly by glycolysis and fatty acid oxidation (72). Cholesterol participates in osteoclast differentiation and multinuclear fusion. Statins and nitrogen-containing bisphosphonates both act on the mevalonate pathway, linking the inhibition of bone resorption to cholesterol biosynthesis at a biochemical level (73).

The membrane role is easy to overlook. Phospholipid acyl chain length and degree of unsaturation influence membrane fluidity, signaling efficiency, and receptor function in membrane microdomains (74). Lipids are also precursors for many lipid-derived signaling molecules.

In metabolic dysregulation the three roles intersect. Accumulating lipid droplets in marrow may fuel osteoblasts, or may mark early lipotoxicity (70). The direction depends on the fatty acid composition of the droplets, on cell type, and on local microenvironment.

### 5.2 Phospholipid peroxidation and ferroptosis

Ferroptosis is a regulated cell death driven by iron-dependent lipid peroxidation. Key elements include polyunsaturated fatty acid (PUFA) phospholipids, the ACSL4–LPCAT3 axis, ALOX15, GPX4, and the Xc^−^–glutathione (GSH) – GPX4 reduction system (75). ACSL4 conjugates PUFA with CoA, and LPCAT3 esterifies the product into membrane phospholipids. Fenton chemistry and lipoxygenases convert these phospholipids into phospholipid hydroperoxides. GPX4 consumes glutathione and reduces the hydroperoxides to the corresponding alcohols (75).

Ferroptosis in bone tissue affects several cell types. In ovariectomized mice, estrogen loss is accompanied by iron deposition in osteoblasts, hypermethylation of the Gpx4 promoter, and GPX4 suppression. The DNMT inhibitor SGI-1027 reverses GPX4 suppression and improves bone loss (76). Under mechanical stress, osteoblast GPX4 falls and ACSL4 rises, with the YAP/TEAD pathway involved (77).

Ferroptosis in aged cortical bone has its own profile. Single-cell transcriptomics of aged mouse cortical bone identified ATF3 as a driver. Senescent osteocytes upregulate ATF3, which raises TfR1-mediated iron uptake and suppresses SLC7A11-mediated cystine import. This combination produces iron overload and lipid peroxidation (78). Targeting ATF3 in aged mice reduces cortical bone ferroptosis and preserves bone mass (78).

Ferroptosis also affects osteoclasts. Oxidative stress, iron overload, SLC7A11, and GPX4 jointly shape the fate of both osteoblasts and osteoclasts (79). Resorption and formation therefore share a common lipid-peroxidation chemistry, but they do not share its direction, and the remainder of this section is written accordingly.

The direction of ferroptosis is not uniform across skeletal cell types, and this section should not be read as evidence that ferroptosis is harmful to bone as a tissue. In osteoblasts, osteocytes, and chondrocytes, ferroptosis is currently best supported as a pathogenic event: GPX4 suppression or ACSL4 induction impairs formation and accelerates cartilage loss, and genetic or pharmacological inhibition is protective in rodent models (76–78, 80, 81); this evidence is graded E3 (animal *in vivo* plus human cell data, no human outcome data). In osteoclasts the sign is reversed or undetermined: these cells tolerate higher reactive oxygen species (ROS) loads, and ferroptosis induction has reduced resorption in some models, so in this lineage the pathway is a candidate therapeutic effector rather than a disease mechanism (75, 79) (E3–E4). In RA, synovial fibroblasts show relative resistance to ferroptosis, which has been proposed as an exploitable vulnerability rather than as a driver of erosive bone loss (82) (E4); we therefore do not treat ferroptosis as a causal contributor to erosive bone damage in RA. The reciprocal relationships between ferroptosis, cellular senescence, and osteoclast homeostasis remain incompletely defined: senescent osteocytes can acquire a ferroptosis-prone phenotype through ATF3-dependent iron handling (78) (E3), but whether ferroptosis sustains, resolves, or merely accompanies the senescent state has not been established in either direction, and we identify this as an open question rather than as a link in the causal chain. Cell-type-resolved directionality, rather than net ferroptotic burden, is therefore the unit of analysis used throughout this Review, and every ferroptosis entry in **Figure 3** and **Table 1** is graded on this basis.

Ferroptosis is the most recently characterized but not the dominant regulated cell-death program in skeletal aging, and presenting it in isolation would overstate its relative contribution. Apoptosis remains the best established: osteocyte apoptosis is the canonical initiating signal for targeted remodeling and is induced by glucocorticoid excess, mechanical unloading, and estrogen withdrawal (25), and the accepted mechanism of action nitrogen-containing bisphosphonates is inhibition of the mevalonate pathway, which prevents prenylation of small GTPases and leads downstream to osteoclast apoptosis (83). One distinction should be made explicit, because this Review applies it throughout: the antifracture efficacy of these agents is E1 in humans, whereas the apoptotic mechanism itself rests largely on preclinical work, and the two should not be graded together. Pyroptosis is next in strength of evidence: NLRP3/caspase-1 activation in estrogen deficiency, diabetic bone, and periodontal models links inflammaging directly to IL-1β release and to loss of osteoblasts and BMSCs (50, 51) (E2–E3), and it is the death program most tightly integrated with the inflammatory node of the present framework. Necroptosis (RIPK1/RIPK3/MLKL) is the least characterized in bone, with scattered evidence in osteoclast survival and chondrocyte death (84) (E4). Ferroptosis currently sits at E3 for osteoblast, osteocyte, and chondrocyte injury and at E3–E4 for osteoclasts, with essentially no human outcome data in any skeletal disease.

These programs are also not mutually exclusive. Lipid peroxidation, inflammasome activation, and mitochondrial stress share upstream inputs including iron availability, ROS, and mitochondrial DNA (mtDNA) release, and most skeletal studies assay a single modality at a single endpoint, so attribution of a phenotype to one death program is frequently underdetermined. Ferroptosis is therefore best framed as one pharmacologically tractable branch of a shared oxidative–inflammatory injury network, notable for cell-type-selective druggability rather than for established primacy. **Table 2** summarizes the comparison.

**Table 2:** Regulated cell-death programs in skeletal aging: relative evidence and directionality.

Program	Key mediators	Best-supported skeletal role	Cell types	Evidence grade	Direction of therapeutic intent
Apoptosis	Caspase-3/9, Bcl-2 family	Osteocyte apoptosis initiates targeted remodeling; osteoclast apoptosis mediates bisphosphonate action	Osteocyte, osteoclast, osteoblast	E1 for clinical antifracture efficacy of agents acting via this route; E2–E3 for the mechanism itself	Inhibit in osteocyte/osteoblast; induce in osteoclast
Pyroptosis	NLRP3, caspase-1, GSDMD, IL-1β	Links inflammaging and estrogen deficiency to osteoblast/BMSC loss and IL-1β release	BMSC, osteoblast, macrophage	E2–E3	Inhibit
Ferroptosis	ACSL4/LPCAT3, GPX4, SLC7A11, iron	Osteoblast/osteocyte/chondrocyte injury; possible suppression of resorption if induced in osteoclasts	Osteoblast, osteocyte, chondrocyte, osteoclast	E3 (E3–E4 in osteoclast; E4 in RA synovial fibroblast)	Cell-type dependent and opposing; requires selective delivery
Necroptosis	RIPK1, RIPK3, MLKL	Scattered evidence in osteoclast survival and chondrocyte death	Osteoclast, chondrocyte	E4	Undetermined

Grades follow the scheme defined in Section 2.2. Upstream inputs are shared across programs, and single-endpoint studies frequently cannot discriminate between them; entries indicate the best-supported role rather than exclusive attribution.

### 5.3 Sphingolipids and S1P signaling

The sphingolipid family is a signaling hub in bone remodeling. The core axis includes ceramide, sphingosine, and sphingosine-1-phosphate (S1P). Their ratio, the so-called sphingolipid rheostat, governs the balance between apoptosis and proliferation (85). Ceramide tends to associate with stress and apoptosis, S1P with survival, migration, and differentiation.

S1P contributes to osteoclast–osteoblast coupling. RANKL stimulates osteoclast precursors and upregulates SPHK1, increasing S1P production. S1P then mobilizes osteoclast precursors away from the bone surface through S1PR1, and produces reverse chemotaxis through S1PR2 (86). S1P also stimulates osteoblast migration and survival, making it a coupling factor between resorption and formation (86).

The role of sphingolipids in inflammatory bone destruction has become clearer. Acid ceramidase is central to regulating the ceramide/S1P ratio. Dysregulation of acid ceramidase promotes inflammatory osteolysis (87).

### 5.4 Saturated fatty acids and ω-3 PUFA

Saturated fatty acids exert lipotoxicity in bone cells. Palmitate activates endoplasmic reticulum (ER) stress, TLR4, and NF-κB, upregulates IL-6 and IL-8, and impairs osteoblast and osteocyte function (88). In BMSCs, long-chain saturated fatty acids inhibit Wnt/β-catenin and BMP2/Runx2 signaling, tipping the differentiation balance toward adipocytes (88).

ω-3 PUFAs (EPA and DHA) act in the opposite direction. A 2023 systematic review of 19 RCTs and 2,546 participants found that ω-3 PUFA supplementation raised blood ω-3 levels but did not change overall BMD or markers such as β-CTX and P1NP. A subgroup analysis suggested a small benefit at the femoral neck in postmenopausal women (89). This evidence is graded E2: randomized human data are available, but the primary endpoint was not met and the subgroup signal is unreplicated.

Mechanistically, the benefit of ω-3 PUFAs partly comes from their downstream lipid metabolites. EPA and DHA are converted by COX, LOX, and CYP450 pathways into resolvins, protectins, maresins, and lipoxins, which together form the specialized pro-resolving mediator (SPM) family. SPMs promote inflammation resolution through dedicated GPCRs (90). In craniofacial and alveolar bone regeneration models, RvE1, MaR1, and LxA4 inhibit osteoclast activity and promote osteoblast proliferation. MaR1 stimulates osteoblast proliferation via the LGR6 receptor (91). Nutritional interventions typically require sustained intake and combine with calcium, vitamin D, and exercise. Standalone ω-3 PUFA supplementation cannot replace established anti-osteoporotic therapy.

### 5.5 Lipid metabolism and BMSC fate decision

BMSC fate decision between osteogenic and adipogenic lineages depends on the balance between PPARγ and Runx2. PPARγ activation promotes adipogenesis, Runx2 activation promotes osteogenesis (92). Lipid environment enters this balance directly. Fatty acids, cholesterol, and oxidized lipids activate PPARγ and push BMSCs toward the adipogenic lineage (69).

Accumulation of BMAT is the tissue-level manifestation of BMSC fate dysregulation. Chemical shift MRI studies show that BMAT increases with age, and the vertebral BMAT in postmenopausal women is particularly elevated (56). BMAT secretes adiponectin and pro-inflammatory factors, and releases free fatty acids into the bone microenvironment through local lipolysis (57).

Glucocorticoid-induced bone loss partly acts through BMAT. Glucocorticoids increase prostaglandin D2-related oxylipins such as 15d-PGJ2 in BMAds, activate PPARγ, trigger BMAd senescence, and propagate this senescence to neighboring cells through SASP (93). Transplantation of senescent BMAds into healthy mouse marrow induces secondary senescence and bone loss; p16-INK4a-deficient BMAds do not produce this effect (93). This establishes a causally transferable link between lipid metabolism, senescence, and bone fragility in mice (E3); the corresponding human evidence is E4, and the inference should not be extended to patients.

### 5.6 Lipid metabolism and immune interface: oxidized lipids as DAMPs

Oxidized phospholipids form a direct interface between lipid metabolism and innate immunity. Under inflammation and oxidative stress, low-density lipoprotein (LDL) is modified into species such as oxidized 1-palmitoyl-2-arachidonoyl-sn-glycero-3-phosphocholine (oxPAPC). These oxidized phospholipids are recognized by TLR2/TLR4, CD14, CD36, and related pattern recognition receptors on macrophages, dendritic cells, endothelial cells, and B-1 cells, where they act as DAMPs and trigger sterile inflammation (94). Oxidized phospholipid DAMPs show bidirectional behavior: at low concentrations they tend to be anti-inflammatory, at high concentrations pro-inflammatory. The net effect depends on cell type and on the surrounding signaling context (94).

In hypercholesterolemic mice, oxidized phospholipids have been shown experimentally to drive inflammation and atherosclerosis (95). This finding causally connects circulating oxidized lipids to systemic chronic inflammation, and serves as a working model of the metabolic–immune interface. Causal evidence for oxidized lipids within the bone microenvironment is currently dominated by animal and *in vitro* studies, and clinical data are limited.

Within bone, oxidized lipids activate PPARγ and push BMSCs toward adipogenesis, and they stimulate osteoblasts to secrete RANKL, which then promotes osteoclastogenesis (69). Immune aging and lipid metabolic dysregulation together push immunoporosis forward, with Th17/Treg imbalance as one of the core mechanisms (63).

Oxidized lipids and inflammaging may form a feedback loop: aged immune cells lose efficient clearance of oxidized lipids and apoptotic cells, and the accumulated oxidized lipids in turn sustain PRR-dependent innate activation (41). The inflammasome arm of this loop is described once only, together with the other regulated cell-death programs, in Section 5.2.1, and is not restated here. The direction and strength of this loop in different tissues and age groups still need more prospective work. From the perspective of bone fragility, this loop may leave older individuals without classical anti-resorptive endogenous regulation, while sustained low-grade inflammation continues to drive osteoclastogenesis.

Most mechanistic evidence cited in this section comes from animal models and *in vitro* studies, and there is a translational gap. Under the scheme defined in Section 2.2, the lipid node is graded predominantly E3, falling to E4 for oxidized-lipid signaling within the human bone microenvironment; it is the least mature of the five nodes examined in this Review. The clinical evidence for ω-3 PUFA and similar nutritional interventions is at a moderate to weak level (89). Drugs targeting ferroptosis or S1P receptors have not yet reached complete Phase III data in bone disease. Whether lipid metabolic markers, including specific oxidized lipid species, circulating S1P, and specific phospholipid composition, can serve as auxiliary biomarkers for bone fragility still awaits validation in prospective cohorts.

Lipid metabolism in bone is not just an energy supply. It enters cell death decisions through phospholipid peroxidation and ferroptosis, mediates osteoclast–osteoblast coupling through sphingolipids and S1P, modulates BMSC direction through the PPARγ–Runx2 axis, and transmits metabolic dysregulation to innate immunity through oxidized lipid DAMPs. Bone fragility can be read as the skeletal manifestation of long-term imbalance at the metabolic–immune interface.

## 6 How converging mechanisms drive bone metabolic abnormalities

### 6.1 Osteoblast–osteoclast uncoupling as a common terminal pathway

OP, OA, RA, PsA, and PDB differ markedly in clinical and radiographic presentation. OP shows systemic bone loss. OA combines cartilage degeneration, subchondral bone remodeling, and synovitis. RA is dominated by erosive bone destruction. PsA shows erosion and ectopic bone formation in the same patient. PDB is focal hyperactive resorption followed by disorganized formation. These diseases share one terminal step: bone resorption and bone formation lose their normal coupling. Consistent with the selection criteria stated in Section 1.1, OP and OA are treated here as aging-driven remodeling disorders and RA and PsA as immune-mediated arthritides in which aging modifies rather than causes the skeletal phenotype. PDB is analyzed as a boundary case, and the respects in which it departs from the shared framework are set out separately in Section 6.4.

The OPG/RANKL ratio is a central parameter for this process. A meta-analysis of 5 RCTs found that the serum OPG/RANKL ratio in OP patients is lower than in controls (27). Osteoblasts in OA subchondral bone show abnormal OPG and RANKL expression, and the altered ratio tracks cartilage degeneration (96). The molecular architecture of the axis is described in Section 3.2 and is not repeated here; this section addresses only the differences between diseases.

Uncoupling can run in two directions. OP and early OA mostly show resorption-over-formation. PDB and some PsA lesions instead show simultaneous elevation of resorption and formation that lose their normal temporal sequence. RA is the prototype of resorption-up and formation-down (97). Mechanistically, IL-6 family cytokines, TNF-α, IL-17, and IL-1 can drive osteoclastogenesis through both RANKL-dependent and RANKL-independent pathways (98).Suppression of Wnt/β-catenin signaling by DKK-1 and sclerostin impairs bone formation in OP and RA (99), but it is not a uniformly shared step: the direction of DKK-1 change is reversed in PsA (Section 6.2). **Figure 2** accordingly represents the Wnt node as two branches of opposite sign rather than as a single shared inhibitory step, and shows RA and PsA as separate skeletal outputs.

Cellular senescence and immunosenescence amplify uncoupling across these diseases. Senescent osteocytes and chondrocytes release SASP factors that keep the local environment in a chronic low-grade inflammatory state (100, 101). The diseases enter from different starting points, but the terminal pathway converges onto the RANKL/OPG axis, Wnt inhibitors, the IL-17/TNF/IL-6 network, and senescence-associated inflammation. The strength of evidence for each disease × mechanism combination is not uniform, and is reported cell by cell in **Figure 3** and **Table 1** using the four-level scheme (E1–E4) defined in Section 2.2.

### 6.2 Cross-disease comparison of shared pathological mechanisms

**Figure 3** compares shared pathological mechanisms across the five disorders. Shading encodes the evidence grade defined in Section 2.2 and a separate symbol encodes mechanistic prominence, because evidence maturity and prominence are distinct properties; cell-level grades and their supporting references are listed in **Supplementary Table 1**. PMOP is primarily driven by estrogen withdrawal and is accompanied by immune skewing, including increased Th17 activity and IL-17 signaling together with reduced Treg-mediated restraint; this imbalance contributes to the osteoimmune basis of PMOP (35) (E2). Age-related osteoporosis places greater emphasis on endogenous senescence-driven inflammation. Senescent osteocytes and stromal/progenitor populations generate a SASP-rich bone microenvironment that favors osteoclastogenesis, impairs osteoblast function, and contributes to delayed skeletal repair (100) (E3). The reduced OPG/RANKL ratio remains the clinically measurable expression of this uncoupling, with the assay limitations set out in Section 3.2.

OA is no longer viewed as a purely mechanical wear-and-tear disorder. The joint is better understood as an integrated organ comprising cartilage, synovium, subchondral bone, and fat pads, in which inflammatory and metabolic signals interact to drive disease progression (102). Early subchondral bone remodeling and disturbance of the OPG/RANKL axis support a bone-remodeling component in OA (96) (E2). Chondrocyte senescence and SASP activity further distinguish OA from simple mechanical injury: senescent chondrocytes release IL-1β, IL-6, and matrix-degrading enzymes, disrupt extracellular matrix (ECM) homeostasis, and can spread senescence-like signals to neighboring cells; senescent-cell clearance mitigates OA progression in experimental models (101) (E3). Synovial macrophage polarization adds another layer, with M1 macrophages releasing TNF-α, IL-1β, and IL-6, whereas M2 macrophages are more closely associated with repair (103). Chondrocyte ferroptosis has also emerged as a relevant mechanism; the JNK-JUN-NCOA4 ferritinophagy axis promotes chondrocyte ferroptosis and worsens OA in experimental models, whereas inhibition of this axis attenuates disease progression (80) (E3). The Wnt–sclerostin–DKK-1 node is engaged in OA as well, but with its sign inverted relative to OP and RA, which is why it is retained in the matrix rather than treated as absent. In human tissue, sclerostin is not confined to osteocytes: it is expressed by articular chondrocytes and is focally increased in the area of cartilage damage in surgically induced animal OA and in end-stage human OA, while being reduced in the subjacent subchondral bone in association with sclerosis (104). Both DKK-1 and sclerostin are detectable in subchondral osteocytes of osteoarthritic femoral heads, with expression highest beneath partial-thickness rather than full-thickness cartilage defects, consistent with an active remodeling front rather than a uniform change (105). *In vivo* perturbation runs in the same direction in both compartments: Sost-null mice subjected to destabilization of the medial meniscus develop higher OA scores with increased aggrecanase and type X collagen expression despite high bone volume (106), and chondrocyte-targeted Dkk-1 overexpression inhibits experimental cartilage destruction by limiting Wnt-driven Mmp13 induction (107) (E2). The therapeutic implication is the reverse of that in OP: where the endogenous inhibitors suppress formation in OP and RA and their blockade is beneficial, in OA they appear to restrain catabolic Wnt activity, so Wnt de-repression of the kind achieved by anti-sclerostin therapy should not be assumed to benefit the osteoarthritic joint. Human interventional evidence exists for the pathway but not for this specific arm: the intra-articular CLK2/DYRK1A inhibitor lorecivivint, which modulates Wnt and inflammatory signaling, improved pain and function and showed effects on medial joint-space width in phase 2b and phase 3 knee OA trials (108, 109), although structural benefit has not been consistently replicated. This cell is accordingly graded E2 · Intermediate: reproducible human tissue evidence together with *in vivo* genetic perturbation satisfies E2, E1 is not reached because the only randomized evidence acts upstream of the sclerostin–DKK-1 arm, and prominence is intermediate rather than high because the node’s effect is distributed between cartilage and subchondral bone rather than dominating either. Clinically, a phase 2a randomized trial in erosive hand OA showed that denosumab 60 mg every 12 weeks improved radiographic structure scores and reduced newly eroded joints at 48 weeks, suggesting that RANKL blockade may be targetable in selected OA subtypes (110) (E2: a single randomized trial with structural rather than fracture endpoints).

RA is typified by synovitis, pannus formation, and juxta-articular erosive bone loss. TNF-α, IL-6, and IL-17 cooperate to induce RANKL expression in synovial fibroblasts and promote Th17 differentiation, thereby linking inflammation to osteoclast-mediated erosion (97) (E1: cytokine-directed therapy retards radiographic progression in randomized trials). A RANKL-independent route has also been described: in RA peripheral blood mononuclear cells, TNF plus IL-6 induced functional bone-resorbing osteoclasts that were not inhibited by OPG, and their abundance correlated with radiographic damage (111) (E3). This finding could help explain residual erosive progression despite RANKL-directed therapy and raises the possibility that part of the bone-protective effect of IL-6R blockade is RANKL-independent. RA also shows suppressed bone formation. In the ESPOIR cohort, higher baseline serum DKK-1 in early RA was associated with radiographic progression over two years (17) (E2), supporting a dual uncoupling pattern of increased resorption and impaired repair.

PsA is distinctive because erosion and ectopic bone formation can occur in parallel. Erosions develop at the bone–pannus interface, whereas enthesophytes, syndesmophytes, and peripheral ankylosis reflect new bone formation (112). The IL-23/IL-17 axis is central to this phenotype: IL-23 promotes Th17 expansion, and IL-17 stimulates stromal and synovial cells to support RANKL-dependent osteoclastogenesis (112) (E2). In contrast to RA, serum DKK-1 is lower in PsA than in RA and healthy controls, potentially releasing Wnt signaling at entheses and contributing to ectopic bone formation (18) (E3). The contrast between the two diseases is one of sign rather than of node membership: the same Wnt–sclerostin–DKK-1 node is engaged in both, but its direction is reversed, and the reversal predicts the divergent skeletal output better than inflammatory intensity does. **Figure 2** has been redrawn on this basis, with the Wnt node split into an upper branch (DKK-1/sclerostin elevation in OP and RA, leading to osteoblast suppression) and a lower branch (relative DKK-1 reduction in PsA, permitting ectopic bone formation), and with RA and PsA shown as separate outputs.

PDB represents focal, disordered, high-turnover remodeling. Lesions contain osteoclasts that are increased in number, larger, and hyperactive, followed by brisk but architecturally disorganized bone formation (3). PDB enters this comparison as a boundary case rather than as a fifth immune-metabolic disease, and is labeled “comparator” in **Figure 3** and **Table 1**. Its genetic and osteocyte-related features, the three respects in which it departs from the shared framework, and the constraint that this places on the convergence claim are set out in Section 6.4 and are not duplicated here.

Cells supported only by indirect or *in vitro* evidence are retained and graded E4 rather than removed, so that gaps in the matrix remain visible. **Figure 3** is therefore an evidence-graded comparative framework rather than a complete causal map, and its grades are ordinal descriptors of study design rather than effect sizes: E1 in one disease is not quantitatively comparable to E1 in another, and no numerical weighting, citation counting, or Delphi procedure was used.

### 6.3 Shared and divergent mechanisms across degenerative bone disease and autoimmune arthritis

Section 6.2, **Figure 3**, and **Table 1** already give the disease-by-disease detail. This section therefore states only the three-layer structure that the comparison yields, and does not restate the individual diseases.

Shared layer. The RANKL/OPG axis is the common terminal pathway, and denosumab improves structural endpoints in both OP and erosive hand OA (110) (E1 in OP, E2 in OA). The TNF-α/IL-6/IL-17 network drives uncoupling in all four non-boundary diseases, whether through RANKL-independent osteoclasts in RA (111), M1-macrophage-led activation in OA (103), or Th17 expansion after estrogen loss in OP (35). Cellular senescence with SASP has been documented in osteocytes in OP, chondrocytes in OA, and lesional osteocytes in PDB (100, 101, 113).

Divergent layer. Divergence is a matter of sign and relative weighting rather than of which nodes are engaged. Degenerative disease is dominated by low-grade systemic inflammation and senescence; autoimmune arthritis by focal, high-intensity adaptive immune activation, with OA intermediate and additionally mechanically driven. The clearest worked example is the Wnt node: DKK-1 is elevated in RA and associated with impaired repair (17), whereas relatively low DKK-1 in PsA is associated with ectopic bone formation (18), so the same node yields opposite skeletal outputs. OA supplies a third variant of the same principle: the node is engaged, but its sign is inverted relative to OP and RA, since the endogenous inhibitors that suppress formation in those diseases appear instead to restrain catabolic Wnt activity in the osteoarthritic joint (104–107). Sign, not membership, again separates the diseases, and here it sets a direct limit on cross-disease repurposing, because anti-sclerostin therapy established in OP cannot be assumed transferable to OA.

Translational layer. Shared nodes make cross-disease repurposing tractable, RANKL blockade in erosive hand OA being the worked example (110); divergence sets its limits, because an intervention that is beneficial where a node runs in one direction may be neutral or harmful where it runs in the other. Ferroptosis-directed therapy is the clearest instance of this constraint (Section 5.2.1). Senolytic strategies have entered clinical evaluation in OP and OA, with bone-density benefit not yet consistent across trials (16) (E3).

Limitations. Most shared pathways rest on animal models and cross-sectional human observation, so causal direction and time course still require prospective and interventional work. Assays for OPG/RANKL, DKK-1, and sclerostin are not standardized, which restricts cross-study comparison (Section 3.2). The framework in this section is intended as a design tool, and the grade attached to each cell of **Figure 3** indicates where it can and cannot yet bear weight.

### 6.4 PDB as a boundary case: where the framework has least reach

PDB is included as a deliberate boundary case rather than as a fifth immune-metabolic disease, and we state plainly that it is the disorder in which the present framework has the least explanatory reach. It deviates in three respects. First, the initiating cause is largely cell-intrinsic and genetic rather than immune: SQSTM1/p62 mutations, most commonly P392L, account for a substantial proportion of familial and a small minority of sporadic cases, mutant p62 enhances NF-κB signaling and perturbs autophagy (3), and the p62P394L knock-in mouse reproduces a PDB-like phenotype (114) (E2); no comparable monogenic driver operates in OP, OA, RA, or PsA. Second, the lesion is focal and only loosely coupled to chronological aging: although prevalence increases with age, systemic inflammaging, Th17/Treg skewing, and lipid-metabolic disturbance have not been shown to be necessary conditions, and direct human evidence for these axes in PDB is E4. Third, the skeletal output is coupled hyperactivity, with resorption and formation both increased but temporally disorganized, rather than the resorption-over-formation imbalance of OP or the erosion-with-impaired-repair pattern of RA.

What PDB contributes is a negative control on the convergence claim. Osteoclast-intrinsic hypersensitivity to RANKL, TNF-α, and 1,25-(OH)_2_D_3_ (3), combined with local osteocyte senescence induced by osteoclast-derived IGF1 (113) (E3), is sufficient to generate a high-turnover phenotype without a systemic immune-metabolic background. If shared-node activation were sufficient rather than permissive, a disease with this presentation should not exist; PDB therefore constrains the framework to a permissive rather than a deterministic reading, which is the sense in which its inclusion is informative. Accordingly, PDB is labeled as a comparator in **Figure 3** and **Table 1**, its immune, senescence, and lipid entries are graded E3–E4, and we do not extend framework-derived therapeutic inferences to PDB beyond RANKL-directed and established antiresorptive treatment.

## 7 Biomarkers and therapeutic targets

### 7.1 Inflammatory and immune-activation biomarkers

Complete blood count-derived inflammatory indices have increasingly been explored in metabolic bone diseases. These include NLR, platelet-to-lymphocyte ratio (PLR), lymphocyte-to-monocyte ratio (LMR), systemic immune-inflammation index (SII, neutrophil count × platelet count/lymphocyte count), systemic inflammation response index (SIRI, neutrophil count × monocyte count/lymphocyte count), and pan-immune-inflammation value (PIV). Their appeal is practical: they are inexpensive, repeatable, and readily available in large-scale screening. A systematic review and meta-analysis of 24 studies found that higher NLR, PLR, SII, SIRI, and PIV were associated with an increased risk of OP (115) (E2: reproducible human observational evidence, without interventional confirmation or a bone-specific mechanistic anchor). In OA, a cross-sectional analysis of NHANES 2005–2018 data reported a positive association between SIRI and adult OA risk (116). A further NHANES-based cohort study of 3,545 adults with OA showed that higher SII and SIRI were independently associated with all-cause and cardiovascular mortality (117). Evidence also supports an association between SII and disease activity in autoimmune diseases, including RA, spondyloarthritis (SpA), and systemic lupus erythematosus (SLE) (118). The major biomarker classes and their translational limitations are summarized in **Table 3**.

**Table 3:** Candidate biomarker classes for immune-metabolic bone disease.

Biomarker class	Representative markers	Main biological signal	Potential use	Current limitation
CBC-derived inflammatory indices	NLR, PLR, LMR, SII, SIRI, PIV	Systemic inflammation and immune-cell balance	Large-scale screening; mortality or risk stratification adjunct	Low tissue specificity; affected by infection, medication and circadian variation
Soluble cytokines	IL-6, TNF-alpha, IL-17A, IL-1beta	Inflammatory activation and osteoclastogenic drive	Mechanism-linked stratification; treatment-response context	Assay variability; weak standalone diagnostic specificity
Bone turnover markers	P1NP, beta-CTX, ALP	Bone formation and resorption dynamics	Treatment monitoring; adherence and early response assessment	Pre-analytical variability; requires standardized timing and interpretation
Senescence markers	p16INK4a, p21Cip1, T-cell p16_variant5	Cellular senescence burden	Trial enrichment for senolytics; aging-related skeletal phenotyping	Cell-type specificity and human assay standardization remain unresolved
SASP composite profile	IL-6, IL-8, CXCL2, CXCL10, MMPs, HMGB1	Senescence-associated inflammatory secretome	Mechanistic phenotyping and senolytic-response assessment	Composite scoring is not yet standardized for routine clinical use
Lipidomic markers	OxPLs, ceramides, sphingolipids, S1P-related species	Metabolic-immune interface and lipid peroxidation	Early disease phenotyping; discovery of therapeutic subgroups	Mostly omics-level evidence; large prospective validation is limited

Biomarker classes are grouped according to biological layer, representative readouts, potential clinical use, and current translational limitations. The table is intended to summarize candidate markers discussed in the review rather than endorse any marker as a standalone diagnostic test. Clinical interpretation requires disease context, assay standardization, sampling conditions, and longitudinal validation. ALP, alkaline phosphatase; CBC, complete blood count; CTX, C-terminal telopeptide of type I collagen; CXCL, C-X-C motif chemokine ligand; HMGB1, high-mobility group box 1; IL, interleukin; LMR, lymphocyte-to-monocyte ratio; MMPs, matrix metalloproteinases; NLR, neutrophil-to-lymphocyte ratio; OxPLs, oxidized phospholipids; P1NP, procollagen type I N-terminal propeptide; PIV, pan-immune-inflammation value; PLR, platelet-to-lymphocyte ratio; SASP, senescence-associated secretory phenotype; SII, systemic immune-inflammation index; SIRI, systemic inflammation response index; S1P, sphingosine-1-phosphate; TNF-α, tumor necrosis factor-α.

The shared limitation of these indices is their low tissue specificity. They are influenced by infection, nutritional status, medication use, and circadian variation. Cut-off values also vary substantially across studies, and these markers have not yet reached the level of standardization required for routine *in vitro* diagnostic use.

IL-6, TNF-α, IL-17A, and IL-1β are more proximal soluble inflammatory mediators in bone remodeling. In a study of postmenopausal women, routine blood testing was used to assess the relationship between inflammatory markers and PMOP. IL-6 increased as bone mineral density declined, and the area under the receiver operating characteristic curve for SII was 0.716, suggesting moderate predictive value (119) (E3: single-cohort discrimination, not externally validated).

Procollagen type I N-terminal propeptide (P1NP), a marker of bone formation, and β-CTX, a marker of bone resorption, are the bone turnover markers recommended by IOF/IFCC. A study including 180 patients with OP and 80 controls showed that β-CTX and P1NP were negatively correlated with BMD, and that higher levels were associated with secondary fracture risk (120). A prospective cohort study further showed that, among fracture patients receiving antiresorptive therapy, P1NP <30 μg/L and β-CTX <0.25 μg/L discriminated treatment adherence and predicted 2-year BMD gains (121) (E2). Compared with dual-energy X-ray absorptiometry (DXA), bone turnover markers respond earlier to treatment and may therefore be useful for early monitoring and treatment adjustment.

### 7.2 Senescence and SASP biomarkers

p16INK4a and p21Cip1 are canonical senescence-associated cell-cycle inhibitors whose expression increases with age in bone and immune cells. Peripheral blood T-cell p16 expression has recently emerged as a clinically accessible marker of senescent cell burden. In a study of postmenopausal women, participants in the highest tertile of T-cell p16_variant5 had a median age of at least 70 years, and T-cell p16 expression correlated with p16 expression in bone biopsies. This supports its use for enriching trial populations most likely to respond to senolytics (122) (E3). p16-positive and p21-positive cells, however, do not mark identical skeletal cell populations. In a mouse fracture model, p21-positive osteochondroprogenitors and neutrophils emerged locally after fracture and impaired repair, whereas age-related bone loss was mainly driven by p16-positive cells (47). This distinction is carried into **Figure 5**, in which the p16 label maps to age-related bone loss and the p21 label to fracture-repair senescence.

Senescent cells release components of the SASP, including IL-6, IL-8, CXCL2, CXCL10, MMPs, and HMGB1. Accumulation of these factors in serum and tissue can serve as a composite readout of senescent burden. A review reported that SASP factors such as IL-6, TNF-α, IFN-γ, and IL-1β regulate RANKL expression and promote osteoclast differentiation, thereby contributing to OP pathogenesis (123) (E3). Compared with a single p16 measurement, composite SASP scores capture a broader secretory phenotype and are now being used to assess responses in senolytic trials (122).

### 7.3 Lipid-metabolism biomarkers

Oxidized phospholipids (OxPLs) can activate inflammation in the bone microenvironment through DAMP signaling and can suppress osteoblast differentiation. In a study of plasma lipoproteins from patients with postmenopausal OP, asymmetrical flow field-flow fractionation coupled with nanoflow UHPLC-ESI-MS/MS revealed marked differences in oxidized phospholipid profiles compared with controls (124). At present, this line of evidence remains largely mechanistic and omics-based, with limited validation in large population cohorts. Clinical translation is therefore still at an early stage, and every entry in this subsection is graded E4 under the scheme defined in Section 2.2: the analytes are not standardized, and no prospective study has related them to a skeletal outcome.

Untargeted lipidomics has also revealed disease-associated serum lipid signatures in OA, OP, and RA. A study including patients with early and late OA and healthy controls found that several serum phospholipid and sphingolipid species were already elevated in early OA (125). At the cartilage level, lipidomic profiling of OA tissue showed ceramide accumulation and reduced odd-chain fatty acid C17:0, with reduced HACL1 activity implicated in cartilage metabolic imbalance (126). Integrated serum metabolomics and lipidomics in 91 patients with RA and 125 healthy controls identified 41 differential metabolites, 25 of which were lipids, with prominent disturbances in sphingolipid metabolism and the urea cycle (127). These three datasets are the whole of the current lipid-biomarker evidence base and are therefore described once, here; Section 8.5 discusses what would be required to move them beyond the marker stage rather than restating them.

### 7.4 Therapeutic strategies

The strategies below are grouped by mechanistic node rather than by disease, and the strength of the evidence supporting each is reported using the scheme defined in Section 2.2. Two scope limits apply. Framework-derived therapeutic inference is not extended to PDB beyond RANKL-directed and established antiresorptive treatment, for the reasons given in Section 6.4. AS is not a target disease of this Review and does not appear in **Figure 3** or **Table 1**; it enters Section 7.4.1 only as therapeutic precedent, and the criteria that exclude it are stated in Section 1.1.

#### 7.4.1 Anti-cytokine therapy

Anti-TNF, anti-IL-6R, and anti-IL-17A therapies are central biologic strategies for RA (128), PsA (129), and AS (130). The RANKL-independent osteoclast route induced by TNF together with IL-6 in RA monocytes (111) is described in Section 6.2 and is not restated here; it provides the rationale for targeting IL-6R and TNF in the control of RA-associated bone destruction. The therapeutic strategies discussed across the Review are summarized in **Table 4**.

**Table 4:** Therapeutic strategies discussed in the review.

Strategy	Representative intervention	Target pathway	Current evidence signal	Key unresolved issue
Anti-cytokine biologics	Anti-TNF, anti-IL-6R, anti-IL-17A	Inflammatory cytokine network	Established in RA/PsA/AS; mechanistic relevance to bone erosion	Disease-specific skeletal endpoints and long-term structural benefit vary
RANKL blockade	Denosumab	RANKL/RANK/OPG axis	Mature antiresorptive therapy; evidence for reduced erosion and improved BMD in selected settings	Rebound risk and sequencing after discontinuation
Wnt pathway modulation	Romosozumab	Sclerostin-Wnt balance	Strong OP fracture and BMD data	Cardiovascular safety, patient selection and sequencing
Senolytics	Dasatinib plus quercetin	Senescent-cell clearance	Phase 2 OP trial supports pathway testing but not consistent BMD benefit	Optimal senescent-cell biomarker, dose schedule and responder subgroup
Senomorphics	Rapamycin, metformin, JAK inhibitors	SASP suppression	Preclinical rationale for reducing inflammatory uncoupling	Human skeletal efficacy and safety windows remain uncertain
Ferroptosis modulation	GPX4-Nrf2/HO-1 axis modulation; deferoxamine	Lipid peroxidation and iron homeostasis	Preclinical benefit in OA and bone-cell injury models	Cell-specific effects and clinical translation in primary OP are unresolved
Nutritional and antioxidant approaches	Omega-3 PUFA, vitamin E	Lipid inflammation and oxidative stress	Mixed or modest effects in meta-analyses and observational studies	Not a substitute for established anti-osteoporotic therapy
MSC/exosome-based therapy	MSC-derived extracellular vesicles	Immunomodulation, osteogenesis and angiogenesis	Promising preclinical OA/OP repair signals	Source heterogeneity, batch control, scaling and prospective clinical data

Representative therapeutic strategies are organized by target pathway, example intervention, current evidence signal, and unresolved translational issue. The table highlights mechanistic and clinical-development directions discussed in the review and should not be read as a treatment guideline or as an indication for off-label use. AS, ankylosing spondylitis; BMD, bone mineral density; GPX4, glutathione peroxidase 4; HO-1, haem oxygenase-1; IL, interleukin; JAK, Janus kinase; MSC, mesenchymal stromal/stem cell; Nrf2, nuclear factor erythroid 2-related factor 2; OA, osteoarthritis; OP, osteoporosis; OPG, osteoprotegerin; PsA, psoriatic arthritis; PUFA, polyunsaturated fatty acid; RA, rheumatoid arthritis; RANK, receptor activator of nuclear factor-κB; RANKL, receptor activator of nuclear factor-κB ligand; SASP, senescence-associated secretory phenotype; TNF, tumor necrosis factor.

Denosumab, which targets RANKL, is an established first-line antiresorptive therapy for OP. A meta-analysis of nine studies involving 1,441 patients with RA showed that denosumab reduced modified total Sharp score, joint-space narrowing, and bone erosion scores while improving BMD (131) (E1). Its structural effect in erosive hand OA rests on a single phase 2a trial and is accordingly graded E2 ( (110); Section 6.2).

Romosozumab, which targets an inhibitor of Wnt signaling, is a bone-forming therapy. The phase 3 FRAME trial enrolled 7,180 postmenopausal women with OP and showed that monthly romosozumab 210 mg for 12 months reduced the risk of new vertebral fracture by 73% compared with placebo (132). In the ARCH trial, conducted in postmenopausal women at high fracture risk, romosozumab followed by alendronate resulted in a lower 24-month incidence of vertebral fracture than alendronate alone (133). A recent meta-analysis of 10 RCTs including 15,476 participants reported greater BMD gains at the lumbar spine, total hip, and femoral neck with romosozumab than with placebo, denosumab, teriparatide, or alendronate (134). These fracture-endpoint trials are the basis on which the OP × Wnt–sclerostin cell of **Figure 3** and **Table 1** is graded E1.

#### 7.4.2 Senolytics and senomorphics

Senolytics act by selectively inducing apoptosis in senescent cells. D+Q is the most extensively studied regimen, and its phase 2 randomized trial in postmenopausal women with OP is described in full in Section 4.4; in summary, the primary β-CTX endpoint was not met, while an exploratory subgroup with high T-cell p16 expression showed a rise in P1NP (16). The trial itself is graded E2 and the subgroup signal E4. Senolytics in OP should therefore still be viewed as pathway-validating interventions rather than established bone-density therapies.

Senomorphics act mainly by suppressing SASP production or weakening downstream SASP effects. Common candidates include rapamycin, metformin, and JAK inhibitors. Reviews suggest that senomorphics can reduce SASP-associated inflammation and uncoupled bone remodeling in animal models, but human evidence remains limited (123) (E3 in animal models; E4 for any skeletal endpoint in humans).

#### 7.4.3 Ferroptosis modulation

Ferroptosis-targeted interventions mainly focus on the GPX4-Nrf2/HO-1 axis and iron homeostasis. A review emphasized the dual role of Nrf2/HO-1 in bone metabolism: moderate activation can protect osteoblasts, whereas excessive activation may aggravate Fenton chemistry through HO-1-mediated Fe^2+^ release (135). Among iron chelators, deferoxamine improved cartilage damage in mouse OA and *in vitro* chondrocyte models by suppressing ferroptosis and activating the Nrf2 pathway (136). Human clinical evidence is currently concentrated in iron-overload-related bone complications, such as those seen in thalassemia, and remains insufficient for primary OP.

A further constraint follows from the cell-type-resolved directionality set out in Section 5.2. Because the sign of ferroptosis differs between the formative lineages and the osteoclast, a systemic modulator cannot be assumed to act in one direction on the skeleton as a whole: GPX4 activation sufficient to protect osteoblasts, osteocytes, and chondrocytes would be expected to preserve osteoclast viability at the same time, and systemic iron chelation such as deferoxamine acts without any lineage selectivity (136). Net skeletal benefit therefore requires cell-selective delivery rather than systemic pathway modulation, and no agent currently meets that requirement. All entries in this subsection are graded E3 or lower, and none has been tested against a human skeletal endpoint.

#### 7.4.4 Nutritional and antioxidant interventions

ω-3 PUFAs are among the most studied nutritional interventions. The evidence is set out in Section 5.4 and is not restated here: supplementation raised circulating ω-3 levels without an overall effect on BMD, β-CTX, N-terminal telopeptide of type I collagen (NTX-1), or CRP, with a subgroup signal at the femoral neck in women or selected populations (89) (E2, primary endpoint not met).

Vitamin E, particularly α-tocopherol, is a lipid-soluble antioxidant that can inhibit lipid peroxidation and ferroptosis. A cross-sectional NHANES-based study reported an inverse association between dietary α-tocopherol intake and OP risk among adults aged 50 years or older (137) (E3). In experimental work, α-tocopherol protected BMSCs from ferroptosis through the PI3K/AKT/mTOR pathway (138) (E4).

#### 7.4.5 Th17/Treg rebalancing

Restoring the Th17/Treg balance is an important direction for interventions that target immune-bone coupling. A review summarized how probiotics, traditional Chinese medicine formulas, and isolated compounds may improve bone remodeling in RA and PMOP by modulating gut ecology and the Th17/Treg ratio (35). A systematic review and meta-analysis of 71 rodent studies found that probiotics increased femoral volumetric BMD (vBMD) and bone volume fraction (BV/TV) in ovariectomized models (139) (E3: reproducible animal evidence without a human skeletal endpoint). Human clinical evidence, however, still comes mainly from small studies, and the optimal strains, dose, and treatment duration remain to be defined.

#### 7.4.6 Mesenchymal stromal cell and exosome-based immunomodulatory therapy

Mesenchymal stem cells (MSCs) and MSC-derived exosomes combine immunomodulatory, osteogenic, and pro-angiogenic effects. BMSC-derived exosomes alleviated OP in an ovariectomized mouse model by promoting M2 macrophage polarization (140). A systematic review of 28 preclinical studies showed that MSC-derived exosomes improved cartilage repair in rat knee OA models, with benefits reflected in OARSI, Mankin, and ICRS scores (141) (E3; E4 for any human skeletal endpoint). MSC-derived extracellular vesicles offer advantages, including avoidance of live-cell toxicity, relative standardizability, and potential for engineering. Their limitations are equally important: source heterogeneity, batch-to-batch variation, and immature scale-up processes continue to constrain clinical translation.

## 8 Key unresolved questions

The coupling between metabolic bone disease and the immune–inflammation–lipid axis has gained substantial empirical support over the past decade. Several core questions remain without definitive answers. This section outlines the open issues and points to where further work is needed. It also locates the three falsifiable predictions derived in Section 1.2. Prediction (i), that senolytic efficacy in OP is conditional on baseline senescent-cell burden rather than uniform across patients, is examined in Section 8.4; prediction (iii), that unselective ferroptosis-directed therapy produces offsetting effects on formation and resorption, in Section 8.2; and prediction (ii), that the direction rather than the absolute concentration of DKK-1 change determines erosive versus ectopic skeletal output, in Section 6.2. Each is stated below together with the study design that would refute it.

### 8.1 Is immune senescence an independent driver of bone loss, or a parallel feature of inflammaging?

Immunosenescence and inflammaging are often discussed together. Whether they drive bone loss independently is still unclear. On the osteoporosis side, the supporting data are reasonably consistent. The “immunoporosis” concept has been used to organize the mechanisms by which aged immune cells contribute to bone fragility (63, 64). At the molecular level, inflammaging appears to be a downstream phenotype of immunosenescence; the two are tightly coupled and the relative weight differs by tissue (142).

Bidirectionality of bone–immune crosstalk makes attribution harder. Aging of the bone marrow microenvironment and peripheral immunosenescence may be mutually reinforcing, so treating immunosenescence as an isolated driver is probably insufficient (143). CD4^+^CD28^−^ and CD8^+^CD28^−^ T cells accumulate with age and promote bone resorption via IL-6, TNF-α and RANKL. Accumulation of senescent T cells in rheumatoid arthritis has been associated with early bone loss (144) (E3).

Evidence in osteoarthritis is weaker. Most reports of immune senescence in OA describe local phenotypic shifts of T cells and macrophages in synovium and subchondral bone, which cannot be equated with systemic immune decline (E4). Observational studies alone will not resolve the question. Separating the contribution of immunosenescence from inflammaging will probably require controlled comparisons of senolytic and anti-inflammatory interventions with bone endpoints.

### 8.2 Does ferroptosis act in opposite directions in osteoblasts and osteoclasts, and can intervention be made cell-selective?

The directional evidence itself, resolved by cell type and graded, is given in Section 5.2, and the comparison with apoptosis, pyroptosis, and necroptosis in Section 5.2.1; this section states what remains unresolved rather than restating that evidence. Osteocyte-specific GPX4 knockout in mice produced bone loss via activation of osteoclast resorption, supporting osteocyte ferroptosis as a target in postmenopausal osteoporosis (145). Osteoclasts tolerate ROS to a greater extent than osteoblasts, and inducing ferroptosis in osteoclasts has reduced bone resorption in some studies (135). The unresolved element is not whether the sign differs, but whether the difference can be exploited pharmacologically.

Cell-selective intervention is difficult in practice. In a D-galactose-induced osteoblast model, YBX1 regulated ferroptosis through the ATF4/FSP1 axis at the level of m5C modification, suggesting cell-type-specific post-transcriptional control points (81). In OA, TfR1 is upregulated in cartilage from DMM mice and in IL-1β-stimulated chondrocytes, and it aggravates inflammation through iron overload and the cGAS/STING pathway; the selective TfR1 inhibitor Ferstatin II reduced cartilage degeneration *in vitro* and *in vivo* (146) (E3). Deferoxamine, by contrast, acts through systemic iron chelation and has no lineage selectivity (Section 7.4.3).

Bone-targeted delivery systems may allow GPX4 activators to accumulate in osteoblasts and osteocytes, or ferroptosis inducers to be released preferentially in hyperactive osteoclasts. Until such delivery is available, balancing ferroptosis inhibition for osteoblast protection against ferroptosis induction for osteoclast suppression remains an open clinical problem. This is prediction (iii) of Section 1.2 in operational form: the claim that net skeletal benefit requires cell-selective delivery would be refuted by demonstration of a net BMD gain with a systemic, non-targeted GPX4 activator.

### 8.3 Can systemic inflammation indices become reliable tools for fracture-risk prediction?

NLR, SII, SIRI and PIV are derived from routine blood counts. Their low cost has driven their use in fracture-risk research. A meta-analysis pooling 8 studies and 3,769 patients reported associations between SII, NLR, PLR, LMR, SIRI and fracture risk in OP populations, with relatively stable pooled effects for SII and NLR (147). A longitudinal cohort study in postmenopausal women with anemia and type 2 diabetes found that elevated SII was independently associated with fragility fracture risk (148). A multicenter prospective study identified SII ≥834.89 as an independent predictor of fracture risk in PMOP (hazard ratio [HR] 3.66, 95% confidence interval [CI] 1.249–10.71) (149) (E2: prospective human data, but no interventional or comparative validation).

Several limitations need to be acknowledged. First, tissue specificity is limited. Systemic inflammation indices are affected by infection, malignancy, cardiovascular events, nutrition and medication, and the bone-specific contribution is hard to isolate. Second, the optimal cut-off varies widely across studies, from values around 600 to above 900, depending on the population, comorbidity profile and analytical platform; cut-offs cannot be freely transposed. Third, the incremental value over the Fracture Risk Assessment Tool (FRAX) has not been demonstrated. Existing studies are mostly single-center and retrospective, with little head-to-head comparison against DXA+FRAX. In a prospective study, the frailty phenotype mediated the association between systemic inflammation and fracture risk, suggesting that part of the observed association may be carried by covarying frailty (150).

For systemic inflammation indices to become reliable predictive tools, large multicenter prospective cohorts, harmonized cut-offs, head-to-head validation against FRAX and tighter control of confounders are still needed.

### 8.4 What are the efficacy and safety windows of senolytics in bone disease?

The concept of senolytics, namely selective clearance of senescent cells, has strong preclinical backing. A 2017 mouse study showed that senolytics prevented age-related bone loss, providing the foundational evidence in this direction (151) (E3). Human evidence is mixed. The 2024 phase 2 RCT of intermittent D+Q in 60 postmenopausal women is reported in full in Section 4.4 and is not restated here (16): the primary β-CTX endpoint was not met, an early and transient P1NP rise was not sustained at 20 weeks, and the responder signal in participants with high T-cell p16 expression came from a *post-hoc* analysis. No serious adverse events were reported, but 20 weeks is a short observation window and longer-term cardiovascular and hematological risks remain unknown. A 2025 review summarized work on natural compounds, kinase inhibitors, Bcl-2 family inhibitors, MDM2/p53 inhibitors and other senolytic classes in osteoporosis, noting a clear gap between preclinical and clinical findings (152).

The safety window raises several specific questions: whether the intermittent D+Q schedule can sustain safety with long-term use; whether senescence-burden heterogeneity needs to be built into entry criteria; whether more selective senolytics can keep efficacy while reducing off-target toxicity. These questions are unlikely to be settled without long-term phase 3 RCTs with multi-endpoint assessment. The second of them is prediction (i) of Section 1.2 in testable form: if senolytic efficacy in OP is conditional on baseline senescent-cell burden, a trial stratified on that burden should show a treatment-by-burden interaction, and the absence of such an interaction would refute the claim.

### 8.5 Are there actionable lipid-metabolism targets in bone disease?

Associations between lipid abnormalities and metabolic bone disease are widely documented at the epidemiological level, but no consensus actionable target has emerged. The three candidate marker sets — oxidized phospholipid profiles in PMOP lipoproteins (124), ceramide accumulation and reduced C17:0 with diminished HACL1 activity in OA cartilage (126), and the sphingolipid and urea-cycle disturbances identified in RA serum (127) — are described in Section 7.3 and are not restated here. What matters for this section is what they do not yet provide: standardized assays, prospective association with a skeletal outcome, and any evidence that modifying the analyte modifies the disease. All three are graded E4. No approved lipid-pathway intervention is currently available for these indications.

An increase in BMAd number is a feature of senile OP. scRNA-seq data suggest that the osteo-adipo fate decision tilts toward adipogenesis with aging, and modulating this decision may be a candidate intervention direction (153) (E3). These avenues remain at the basic-research stage, with a considerable distance to clinical candidates. An actionable target would need to satisfy three conditions that none currently meets: a standardized human assay, a demonstrated prospective relationship to fracture or structural progression, and a tractable intervention point that is not shared with an essential systemic lipid function.

## 9 Conclusions and outlook

### 9.1 Immune–metabolic convergence as an organizing framework: what is and is not claimed

Over the past decade, research on aging-driven remodeling disorders, immune-mediated arthritis and metabolic bone disease has converged on a shared framework: the immune–inflammation–metabolism axis is a central regulator of bone homeostasis. We do not claim to have identified new mechanisms. The convergence described here is stated as four specific claims rather than as a general assertion (Section 1.2): the convergence set is enumerated as five nodes; disease identity follows from the sign and weighting of node activity rather than from which nodes are engaged, DKK-1 in RA versus PsA being the worked example; the lipid–ferroptosis axis is brought inside the node set with its direction resolved by cell type; and every disease × mechanism cell is graded. Several layers of evidence support this view. At the cellular level, senescence and functional drift in T cells, B cells, macrophages and neutrophils reshape the osteoblast–osteoclast balance (142, 144). At the molecular level, SASP factors, RANKL, IL-6, TNF-α and IL-17A form pathways shared across diseases. At the metabolic level, oxidized phospholipids, ceramides and lipid peroxidation products from ferroptosis act as DAMPs that link metabolic disturbance to immune activation (124, 135). The lipid arm carries the cell-type-resolved direction of Section 5.2 rather than a general claim that ferroptosis harms bone: pathogenic in osteoblasts, osteocytes and chondrocytes (E3), reversed or undetermined in osteoclasts (E3–E4), and one tractable branch of a shared oxidative–inflammatory network rather than the dominant cell-death program in bone, which on present evidence remains apoptosis, with pyroptosis most tightly coupled to the inflammatory node (50, 51) and necroptosis least characterized in skeletal tissue (84) (Section 5.2.1, **Table 2**). Treating RA and OP as paired prototypes of immunosenescence in osteoimmunology fits this cross-disease framework (153).

Placing immune processes at the center does not deny the role of bone-resident cells, endocrine signals or mechanical loading. The framework rebalances the hierarchy used to interpret pathogenesis. It is also stated at a defined level of confidence: every disease × mechanism combination carries a grade from E1 to E4 on the scheme defined *a priori* in Section 2.2, reported cell by cell in **Figure 3** and **Table 1**. The grades are ordinal descriptors of study design, not effect sizes, and no numerical weighting, citation counting or Delphi procedure was used. The clinical implication is that combination interventions across nodes of the immune–inflammation–metabolism axis may be more useful than single-target attacks, a proposition that the three falsifiable predictions set out in Section 1.2 are intended to test rather than to assume.

### 9.2 Boundary conditions: where the framework has least reach

The framework is permissive rather than deterministic, and PDB marks the limit of its reach. PDB is retained as a comparator rather than as a fifth immune-metabolic disease (Sections 1.1 and 6.4), and deviates in three respects: its initiating mechanism is largely cell-intrinsic and monogenic (3, 114); its lesion is focal and only loosely coupled to chronological aging, with human evidence for the inflammaging, Th17/Treg and lipid axes graded E4; and its output is coupled hyperactivity rather than uncoupling, generated by osteoclast-intrinsic hypersensitivity with local osteocyte senescence (113) and no systemic immune-metabolic background. A disorder of this kind should not exist if shared-node activation were sufficient rather than permissive, which is why we do not extend framework-derived therapeutic inference to PDB beyond RANKL-directed and established antiresorptive treatment. Two limits apply more broadly. This is a narrative, mechanism-oriented synthesis rather than a systematic review (Section 2.1): the search is auditable, but selection was purposive and selection bias cannot be excluded. And convergence at the level of nodes does not license convergence at the level of therapy, since the same node may require opposite manipulation in different lineages.

### 9.3 An integrated view of treatment strategies

Re-reading current therapies through this immune-driven lens suggests a three-tier view. The first tier consists of mature antibody drugs targeting RANKL (denosumab), TNF-α, IL-6R, IL-17A and sclerostin (romosozumab). These agents have substantial RCT support across RA, OP and PsA, and in ankylosing spondylitis, which is not analyzed as a target disease here and is cited only as therapeutic precedent (Sections 1.1 and 7.4.1), and they act by blocking key nodes of immune–bone coupling. The second tier covers early-clinical interventions on aging and metabolic pathways: senolytics, senomorphics, ferroptosis modulators and nutrition–antioxidant strategies. Here the preclinical and clinical pictures diverge (16, 152), and subgroup heterogeneity is large; biomarker-stratified trial designs are likely needed. For the ferroptosis arm, the opposing cell-type directionality of Section 5.2 implies that net skeletal benefit requires cell-selective delivery rather than systemic pathway blockade; in RA, the relative ferroptosis resistance of synovial fibroblasts is better read as an exploitable vulnerability than as a driver of erosion (82). The third tier covers regenerative and immunomodulatory strategies, such as MSCs, MSC-derived exosomes, engineered nanocarriers, and bone-targeted drug delivery systems; most of these remain in preclinical or early human exploration. The tiers track evidential maturity rather than expected effect size: first-tier targets rest on E1–E2 evidence, second-tier interventions on E3, and third-tier strategies largely on E3–E4.

Cross-tier combination strategies may be the main future direction. Romosozumab followed by denosumab as a BMD consolidation regimen has entered clinical use; senolytic pre-treatment followed by bone-forming agents has shown additive effects in animal models (152); the phase 2 senolytic trial that anchors this expectation is described once, in Section 4.4, and is not restated here.

### 9.4 Emerging analytical approaches

Three analytical developments bear on the questions above and are noted in outline rather than reviewed in detail. Multi-omics integration serves to locate causal nodes rather than to generate further markers: sphingolipid metabolism and the urea cycle in RA (127), and oxidized phospholipid signatures in PMOP lipoproteins (124). Single-cell and spatial transcriptomics place those nodes in tissue context, identifying the osteo-adipo fate shift in senile OP (154) and TGF-β as a regulator of osteoblast activation on quiescent surfaces, where dual TGF-β and sclerostin inhibition outperformed sclerostin inhibition alone (155). Machine learning has reached a pooled mean AUC of 0.925 for fragility-fracture prediction across 13 studies and 24,489 patients, exceeding DXA and FRAX used alone (156); individual model-development studies and reviews are consistent with this (157–160). All three depend on assay standardization, model interpretability and population diversity, and the regulatory and validation framework for AI in OP management remains incomplete (160).

### 9.5 A realistic path to clinical translation

Translating these technologies and mechanistic insights into clinical practice is not a linear path. In the short term (3–5 years), plausible translational directions include composite fracture-prediction models combining systemic inflammation indices with bone turnover markers entering guidelines; senolytic trials with senescence-burden stratification advancing to phase 3; and regulatory clarification for MSC-derived exosomes in local OA injection. The second of these is prediction (i) of Section 1.2 in trial form: if senolytic benefit in OP proves uniform rather than conditional on baseline senescent-cell burden, the stratified design becomes unnecessary and that component of the framework fails. In the medium term (5–10 years), bone-targeted nanocarrier delivery, cell-selective ferroptosis modulation and AI-driven opportunistic CT screening for BMD may enter mainstream practice. Cell selectivity is the operative qualifier: what distinguishes ferroptosis from the better-characterized death programs is selective druggability, not established primacy (**Table 2**). Over the longer term (>10 years), a precision bone–immune medicine framework integrating multi-omics, single-cell, spatial-omics and AI may gradually displace the current BMD-centered model. These timelines are projections and may shift with technology maturation, regulatory developments and data availability.

Several coordinated efforts will be needed. Standardization of clinical assays is foundational; consistent SII cut-offs, p16 detection methods and SASP scores across laboratories are prerequisites for pooling clinical evidence. Multicenter prospective RCTs are central, particularly in senolytics, ferroptosis modulation and MSC-EV areas. Romosozumab, as a Wnt–sclerostin-targeting drug that has moved from mechanism studies through phase 3 RCTs to guideline incorporation, offers a workable template (132, 133), and the OP × Wnt–sclerostin combination is graded E1 on that basis in **Figure 3** and **Table 1**.

The framework will be useful in proportion to how readily it can be shown to be wrong; the three predictions of Section 1.2, the graded cells of **Figure 3** and **Table 1**, and the boundary case of Section 6.4 are offered in that spirit.

## Glossary

ACSL4: acyl-CoA synthetase long-chain family member 4
AS: ankylosing spondylitis
ASAS-EULAR: Assessment of SpondyloArthritis international Society–European Alliance of Associations for Rheumatology
ATF3: activating transcription factor 3
BMAT: bone marrow adipose tissue
BMD: bone mineral density
BMP: bone morphogenetic protein
BMSC: bone marrow stromal/stem cell
CI: confidence interval
CRP: C-reactive protein
CTX: C-terminal telopeptide of type I collagen
D+Q: dasatinib plus quercetin
DAMPs: damage-associated molecular patterns
DHA: docosahexaenoic acid
DKK-1: Dickkopf-1
DXA: dual-energy X-ray absorptiometry
EPA: eicosapentaenoic acid
ER: endoplasmic reticulum
EULAR: European Alliance of Associations for Rheumatology
EV: extracellular vesicle
FRAX: Fracture Risk Assessment Tool
GCA: grancalcin
GPX4: glutathione peroxidase 4
GSH: glutathione
HR: hazard ratio
IFN-γ: interferon-γ
IL: interleukin
ITAM: immunoreceptor tyrosine-based activation motif
LDL: low-density lipoprotein
LMR: lymphocyte-to-monocyte ratio
LPCAT3: lysophosphatidylcholine acyltransferase 3
M-CSF: macrophage colony-stimulating factor
MMP: matrix metalloproteinase
MSC: mesenchymal stromal cell
mtDNA: mitochondrial DNA
NF-κB: nuclear factor-κB
NK: natural killer
NLR: neutrophil-to-lymphocyte ratio
NLRP3: NOD-like receptor family pyrin domain-containing 3
OA: osteoarthritis
OP: osteoporosis
OPG: osteoprotegerin
oxPAPC: oxidized 1-palmitoyl-2-arachidonoyl-sn-glycero-3-phosphocholine
P1NP: procollagen type I N-terminal propeptide
PDB: Paget’s disease of bone
PLR: platelet-to-lymphocyte ratio
PMOP: postmenopausal osteoporosis
PPARγ: peroxisome proliferator-activated receptor-γ
PsA: psoriatic arthritis
PUFA: polyunsaturated fatty acid
RA: rheumatoid arthritis
RANKL: receptor activator of nuclear factor-κB ligand
RCT: randomized controlled trial
ROS: reactive oxygen species
Runx2: runt-related transcription factor 2
S1P: sphingosine-1-phosphate
SASP: senescence-associated secretory phenotype
SII: systemic immune-inflammation index
SIRI: systemic inflammation response index
SLC7A11: solute carrier family 7 member 11
SPHK1: sphingosine kinase 1
SPM: specialized pro-resolving mediator
STING: stimulator of interferon genes
TfR1: transferrin receptor 1
TGF-β: transforming growth factor-β
Th17: T helper 17 cell
TLR: Toll-like receptor
TNF-α: tumor necrosis factor-α
Treg: regulatory T cell
