# Supplementary material for: Immune–metabolic convergence in skeletal remodeling disorders: shared osteoimmune, senescent and lipid mechanisms across aging-related and immune-mediated bone disease
Source: Front Immunol. 2026 Sep 25;17:1949165. doi: 10.3389/fimmu.2026.1949165 (PMC13650773; doi:10.3389/fimmu.2026.1949165)
Supplement: Supplementary file 1 [file Table1.docx]

**Supplementary**

**Table S1 | Cell-level evidence grades, mechanistic prominence and supporting references for the disease × mechanism matrix (Figure 5; Table 1).**

| **Cell** | **Disease** | **Mechanistic node** | **Grade** | **Prominence** | **Supporting references** | **Basis for the grade** | **Section(s)** |
| --- | --- | --- | --- | --- | --- | --- | --- |
| **OP-1** | OP | RANKL/OPG | **E1** | High | 26, 27, 30, 65 | Pooled randomized evidence for denosumab on bone mineral density and bone turnover endpoints; reproducible human OPG/RANKL cohorts with assay heterogeneity | 3.2, 4.4, 6.1, 6.2, 7.4.1 |
| **OP-2** | OP | IL-17/TNF-α/IL-6 | **E2** | Intermediate | 31, 35, 36, 38 | Reproducible human Th17/Treg observation together with animal perturbation | 3.3–3.5, 6.2 |
| **OP-3** | OP | SASP/inflammaging | **E2** | High | 11, 16, 43, 44, 65, 66, 100 | Randomized phase 2 senolytic trial powered on a turnover marker; primary endpoint not met (subgroup signal E4) | 4.1–4.5, 6.2, 7.4.2 |
| **OP-4** | OP | Lipid peroxidation/ferroptosis | **E3** | Low | 76, 77, 78, 139 | Rodent in vivo GPX4, ACSL4 and ATF3 perturbation; no human outcome data | 5.2, 7.3, 8.2 |
| **OP-5** | OP | Wnt–sclerostin–DKK-1 | **E1** | High | 99, 126, 127, 128 | Phase 3 romosozumab trials with vertebral fracture endpoints | 6.1, 7.4.1, 9.5 |
| **OA-1** | OA | RANKL/OPG | **E2** | Intermediate | 96, 104 | Human subchondral OPG/RANKL data plus a phase 2a randomized trial with structural endpoints | 6.1, 6.2, 7.4.1 |
| **OA-2 †** | OA | IL-17/TNF-α/IL-6 | **E3** | Intermediate | 12, 15, 102, 103 | Synovial macrophage and cytokine data without a cytokine-directed structural-endpoint trial | 6.2, 6.3 |
| **OA-3** | OA | SASP/inflammaging | **E3** | High | 6, 101 | Senescent-cell clearance mitigates experimental OA; no human outcome data | 6.2, 6.3 |
| **OA-4** | OA | Lipid peroxidation/ferroptosis | **E3** | Intermediate | 80, 119, 120, 130, 140 | Animal in vivo ferritinophagy and TfR1 evidence; lipidomic markers alone remain E4 | 5.2, 7.3, 8.2 |
| **OA-5 †** | OA | Wnt–sclerostin–DKK-1 | **E4** | Low | — | No OA-specific evidence retrieved; cell retained so that the gap remains visible | 6.2, Table 1 |
| **RA-1** | RA | RANKL/OPG | **E1** | High | 97, 98, 105, 125 | Denosumab meta-analysis with Sharp-score and erosion endpoints | 6.1–6.3, 7.4.1 |
| **RA-2** | RA | IL-17/TNF-α/IL-6 | **E1** | High | 38, 40, 97, 105, 122 | Cytokine-directed therapy retards radiographic progression in randomized trials | 3.5, 6.2, 7.4.1 |
| **RA-3** | RA | SASP/inflammaging | **E3** | Intermediate | 138 | Senescent T-cell accumulation associated with early bone loss; single cohort | 8.1, 9.1 |
| **RA-4** | RA | Lipid peroxidation/ferroptosis | **E4** | Low | 82 | Synovial fibroblast ferroptosis resistance framed as an exploitable vulnerability, not as a driver of erosion | 5.2, 7.3, 9.3 |
| **RA-5** | RA | Wnt–sclerostin–DKK-1 | **E2** | High | 17, 99 | ESPOIR cohort: higher baseline DKK-1 predicts 2-year radiographic progression | 6.2, 6.3 |
| **PsA-1** | PsA | RANKL/OPG | **E2** | Intermediate | 106 | Entheseal RANKL-dependent osteoclastogenesis; human observational evidence | 6.2 |
| **PsA-2** | PsA | IL-17/TNF-α/IL-6 | **E2** | High | 106, 123 | IL-23/IL-17 axis mechanistically established; no structural-endpoint trial cited in this Review | 6.2, 7.4.1 |
| **PsA-3 †** | PsA | SASP/inflammaging | **E4** | Low | — | Possible local amplifier only; no PsA-specific evidence retrieved | 6.2, Table 1 |
| **PsA-4 †** | PsA | Lipid peroxidation/ferroptosis | **E4** | Low | — | No PsA-specific evidence retrieved; cell retained so that the gap remains visible | 6.2, Table 1 |
| **PsA-5** | PsA | Wnt–sclerostin–DKK-1 | **E3** | High | 18 | Serum DKK-1 lower than in RA and healthy controls; single unreplicated human study | 6.2, 6.3, 9.1 |
| **PDB-1** | PDB | RANKL/OPG | **E2** | High | 3, 108 | Osteoclast-intrinsic hypersensitivity plus p62 P394L knock-in phenocopy in mice | 6.2, 6.4 |
| **PDB-2 †** | PDB | IL-17/TNF-α/IL-6 | **E4** | Low | 3 | Heightened osteoclast responsiveness only; direct human evidence stated as E4 in Section 6.4 | 6.4 |
| **PDB-3** | PDB | SASP/inflammaging | **E3** | Intermediate | 107 | Osteoclast-derived IGF1 induces local osteocyte senescence | 6.2, 6.4 |
| **PDB-4 †** | PDB | Lipid peroxidation/ferroptosis | **E4** | Low | — | No PDB-specific evidence retrieved; cell retained so that the gap remains visible | 6.4, Table 1 |
| **PDB-5 †** | PDB | Wnt–sclerostin–DKK-1 | **E4** | Low | — | No PDB-specific evidence retrieved; cell retained so that the gap remains visible | 6.4, Table 1 |

Note: Grades follow the four-level scheme defined a priori in Section 2.2 and were assigned independently by two authors on the basis of the highest available evidence tier, with disagreements adjudicated by the third author. Prominence (High/Intermediate/Low) is graded separately from evidence level. † indicates a cell for which no disease-specific evidence meeting the inclusion criteria was retrieved, or for which the available evidence is indirect; these cells are graded E4 and retained rather than removed so that gaps in the field remain visible.
