# Supplementary material for: Immune–metabolic convergence in skeletal remodeling disorders: shared osteoimmune, senescent and lipid mechanisms across aging-related and immune-mediated bone disease
Source: Front Immunol. 2026 Sep 25;17:1949165. doi: 10.3389/fimmu.2026.1949165 (PMC13650773; doi:10.3389/fimmu.2026.1949165)
Supplement: Supplementary file 2 [file Table2.docx]

**Supplementary Methods S1**

# S1.1 Databases and search window

PubMed/MEDLINE, Embase and the Web of Science Core Collection were searched from 1 January 2000 to 31 July 2026, supplemented by ClinicalTrials.gov for interventional evidence. This is a narrative, mechanism-oriented review; it is not registered as a systematic review, does not claim exhaustive coverage, and no PRISMA flow diagram is presented. Retrieval was iterative and purposive rather than executed as a single pre-specified query, so no record counts are reported at each stage; selection bias arising from this approach is mitigated by grading every disease × mechanism claim (Section 2.2 of the main text) and by retaining and flagging weakly supported entries rather than omitting them.

# S1.2 Concept blocks and search terms

Search strings combined four concept blocks with Boolean operators. Controlled-vocabulary terms (MeSH in PubMed/MEDLINE, Emtree in Embase) were used where a heading exists for the concept; title/abstract free text was used for concepts that have none. The full term list, by block, is given in Table S1.1.

## Block (i) — the five disorders

| **Controlled vocabulary (MeSH/Emtree)** | **Free text** |
| --- | --- |
| Osteoporosis; Osteoporosis, Postmenopausal; Osteoarthritis; Arthritis, Rheumatoid; Arthritis, Psoriatic; Osteitis Deformans | — (all six disorders map to a controlled-vocabulary heading; Osteitis Deformans is the indexed heading for Paget's disease of bone) |

## Block (ii) — the five mechanistic nodes of the framework

| **Node** | **Controlled vocabulary (MeSH/Emtree)** | **Free text** |
| --- | --- | --- |
| RANKL/OPG axis | RANK Ligand; Osteoprotegerin | RANK |
| IL-17/TNF-α/IL-6 network | — | interleukin-17; tumor necrosis factor-alpha; interleukin-6; interleukin-23; Th17 cells; regulatory T cells |
| SASP/inflammaging | Cellular Senescence; Immunosenescence | senescence-associated secretory phenotype; inflammaging; p16INK4a; p21 |
| Lipid peroxidation/ferroptosis | Ferroptosis; Lipid Peroxidation | GPX4; ACSL4; oxidized phospholipid; sphingosine-1-phosphate; bone marrow adipose tissue |
| Wnt–sclerostin–DKK-1 balance | — | Wnt; sclerostin; Dickkopf-1 |

Terms for the remaining regulated cell-death programs — apoptosis, pyroptosis, NLRP3, necroptosis — were included as free text so that ferroptosis could be weighted against them rather than presented in isolation (see main text, Section 5.2.1 / Table 4). Two cross-cutting free-text terms without a controlled-vocabulary heading, osteoimmunology and immunoporosis, and one cell-lineage term, osteomorph, were also searched across Block (ii) rather than restricted to a single node.

## Block (iii) — interventions directed at those nodes

Free text: denosumab; romosozumab; bisphosphonate; anti-TNF; interleukin-6 receptor blockade; interleukin-17 inhibition; senolytic; dasatinib; quercetin; senomorphic; iron chelation; deferoxamine; n-3 polyunsaturated fatty acid; vitamin E; probiotics; mesenchymal stromal cell; extracellular vesicle.

## Block (iv) — biomarker and skeletal outcome terms

Free text: bone mineral density; fracture; bone turnover marker; P1NP; C-terminal telopeptide; neutrophil-to-lymphocyte ratio; systemic immune-inflammation index; lipidomics; metabolomics; single-cell RNA sequencing; machine learning.

## Additional sources

Reference lists of retrieved reviews and forward citations of key mechanistic papers were screened manually. Current European Alliance of Associations for Rheumatology (EULAR) and Assessment of SpondyloArthritis international Society–EULAR (ASAS-EULAR) management recommendations, and Global Burden of Disease estimates, were retrieved directly to support the epidemiological statements in Section 1 and the therapeutic-precedent statements in Section 7.4 of the main text.

# S1.3 Eligibility criteria

## Inclusion criteria

• Publications in English reporting primary studies, interventional trials or systematic reviews with a bone, cartilage or immune-cell outcome in one of the five selected disorders, or in an accepted model thereof.

• Mechanistic studies characterizing one of the five framework nodes in a skeletal or joint cell type.

## Exclusion criteria

• Case reports.

• Conference abstracts without a peer-reviewed full text.

• Studies without an explicit skeletal or articular readout.

• Studies reporting bone mineral density alone, without a stated mechanistic hypothesis.

# S1.4 Screening procedure

• Titles and abstracts were screened by two authors independently against the eligibility criteria in S1.3.

• Full texts of records passing that first stage were assessed in duplicate by the same two authors.

• Disagreements at either stage were resolved by discussion with the third author.

This procedure is the same one summarised in Section 2.1 of the main text; it is given here in full for completeness.
